# Ultrathin Films of MXene Nanosheets Decorated by Ionic Branched Nanoparticles with Enhanced Energy Storage Stability

**DOI:** 10.1021/acsami.3c09064

**Published:** 2023-11-07

**Authors:** Paraskevi Flouda, Alex Inman, Mariana Gumenna, Daria Bukharina, Valery V. Shevchenko, Yury Gogotsi, Vladimir V. Tsukruk

**Affiliations:** † School of Materials Science and Engineering, Georgia Institute of Technology, Atlanta, Georgia 30332, United States; ‡ A. J. Drexel Nanomaterials Institute and Department of Materials Science and Engineering, Drexel University, Philadelphia, Pennsylvania 19104, United States; § Institute of Macromolecular Chemistry of the National Academy of Sciences of Ukraine, Kharkivske Shosse 48, Kyiv 02160, Ukraine

**Keywords:** modified MXenes, polyhedral oligomeric silsesquioxanes, ultrathin supercapacitor, long-term energy storage stability

## Abstract

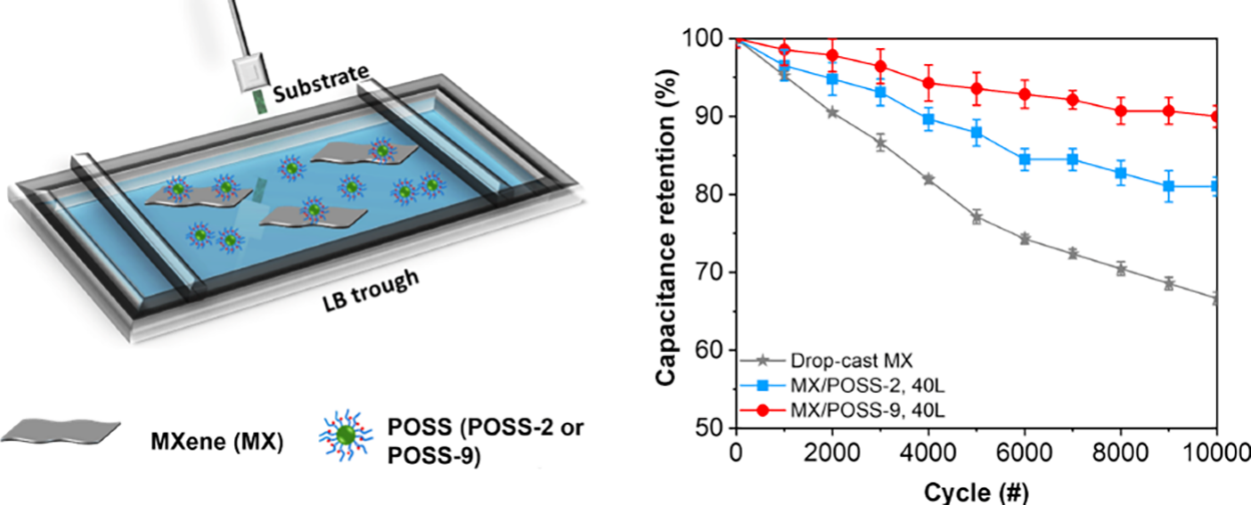

Two-dimensional (2D) materials such as MXenes have shown great potential for energy storage applications due to their high surface area and high conductivity. However, their practical implementation is limited by their tendency to restack, similar to other 2D materials, leading to a decreased long-term performance. Here, we present a novel approach to addressing this issue by combining MXene (Ti_3_C_2_T_*x*_) nanosheets with branched ionic nanoparticles from polyhedral oligomeric silsesquioxanes (POSS) using an amphiphilicity-driven assembly for the formation of composite monolayers of nanoparticle-decorated MXene nanosheets at the air–water interface. The amphiphilic hybrid MXene/POSS monolayers allow for the fabrication of organized multilayered films with ionic nanoparticles supporting the nanoscale gap between MXene nanosheets. For these composite multilayers, we observed a 400% enhancement in specific capacitance compared to pure drop-cast MXene films. Furthermore, dramatically enhanced electrochemical cycling stability for ultrathin-film electrodes (<400 nm in thickness) with a 91% capacitance retention over 10,000 cycles has been achieved. Our results suggest that this insertion of 0D ionic nanoparticles with complementary interactions in between 2D MXene nanosheets could be extended to other hybrid 0D–2D nanomaterials, providing a promising pathway for the development of hybrid electrode architectures with enhanced ionic transport for long-term energy cycling and storage, capacitive deionization, and ionic filtration.

## 1 Introduction

The increasing demand for portable, flexible, and wearable electronics has led to an increased need for miniaturized, thin, and lightweight energy storage devices.^1,2^ Two-dimensional (2D) materials can form flexible and mechanically robust films with high packing density that can meet the requirements of flexible and wearable electronics. Various 2D nanomaterials, such as reduced graphene oxide (rGO), metal oxides, and MXenes, have been considered as active electrode materials using a variety of different fabrication techniques, including layer-by-layer (LbL) methods, spin-coating, spray-coating, and vacuum-assisted filtration.^2−4^ Among others, rapidly emerging 2D nanomaterials, MXenes, are promising candidates for energy storage applications due to their high electrical conductivity (up to 20,000 S/cm for Ti_3_C_2_T_*x*_), high surface area, and pseudocapacitive behavior, leading to enhanced capacitance values.^5−7^ Specifically, MXenes represent a family of 2D transition metal carbides, nitrides, and carbonitrides with a general formula of M_*n*+1_X_*n*_T_*x*_, where M is an early transition metal, X is carbon and/or nitrogen, and T_*x*_ is the surface terminal group (e.g., −O, −OH, −F, and/or −Cl), with *n* = 1, 2, 3, or 4 and *x* representing the number of functional groups per unit.^8^ As known, MXenes are typically synthesized using a top-down method by selective etching of A layers from layered ternary nitrides/carbide MAX phases, where A represents an element from group IIIA or IVA of the periodic table.^6^

Owing to their exceptional properties, MXenes have been utilized in electrodes for various electrochemical energy storage devices, including supercapacitors, lithium-ion batteries, and sodium-ion batteries.^9,10^ For supercapacitors specifically, free-standing pure Ti_3_C_2_T_*x*_ film electrodes of 5 μm in thickness demonstrated capacitance values of ∼455 mF/cm^2^ (910 F/cm^3^) at 2 mV/s with an aqueous 1 M H_2_SO_4_ electrolyte.^11,12^ Similarly, Ti_3_C_2_T_*x*_ air-sprayed electrodes of 50 nm on gold substrates exhibited capacitance values of 7.5 mF/cm^2^ (1500 F/cm^3^) at 10 mV/s using a PVA/H_3_PO_4_ gel electrolyte.^7,13^ For these materials, it has been suggested that restacking arising from strong hydrogen bonding interactions between the adjacent nanosheets hinders the electrolyte ion movement within the electrodes, decreasing capacitance values, rate capability, and cycling stability with increasing electrode thickness.^14^ To avoid these issues, the addition of nanomaterials that act as spacers between the MXene flakes, such as rGO, carbon nanotubes (CNTs), and various nanoparticles, has been suggested.^14^ For example, free-standing Ti_3_C_2_T_*x*_/rGO electrodes exhibited capacitance values of 1040 F/cm^3^ at 2 mV/s.^15^ Similarly, LbL electrodes of Ti_3_C_2_T_*x*_/positively charged functionalized Ti_3_C_2_T_*x*_ (f-Ti_3_C_2_T_*x*_) of 63 nm in thickness with a 1 M H_2_SO_4_ electrolyte at 10 mV/s demonstrated capacitance values of 13 mF/cm^2^ (2080 F/cm^3^).^16^

On the other hand, as known, very small (1–2 nm) and relatively uniform polyhedral oligomeric silsesquioxane (POSS) nanoparticles are composed of Si–O cage-like cores with well-defined structures and an organic shell with functional groups, such as ionic moieties or chromophores.^17−19^ The introduction of POSS nanoparticles in free-standing rGO-based electrodes (30 μm thick) has been explored for the improvement of capacitance values (350 mF/cm^2^, 115 F/cm^3^ at 1 mV/s with 1 M H_2_SO_4_), thus doubling the capacitance of pure rGO.^20^ The improvements in energy storage were attributed to the expansion of the interlayer spacing of the rGO nanosheets and the moderate POSS pore sizes that accommodate electrolyte ion diffusion.^20^

Here, we report novel multilayered supercapacitor MXene/POSS ultrathin films (<400 nm in thickness) as electrodes with improved cycling stability compared to their single-component MXene counterpart. We demonstrate that the introduction of ionic branched amphiphilic POSS nanoparticles with variant peripheral composition stabilizes the MXene nanosheets at the air–water interface and allows for the fabrication of hybrid monolayers with MXenes nanosheets decorated with ionic POSS nanoparticles. Indeed, atomic force microscopy (AFM) revealed the formation of monolayers composed of micellar POSS nanoscale domains in between the MXene nanosheets depending on POSS peripheral hydrophilic/hydrophobic balance. The addition of ionic POSS nanoparticles not only stabilizes the MXenes at the air–water interface but also facilitates significantly improved energy storage performance and stability. The capacitance values of 17 mF/cm^2^ and high capacitance retention values up to 91% after 10,000 cycles are significantly higher than those for the pure MXene film. We suggest that POSS nanoparticles are placed in between the multilayers and hinder MXene restacking. Furthermore, the formation of POSS-supported channels facilitates the enhanced ion transport across loosely packed 2D MXene nanosheets. Overall, this work provides a route for designing hybrid ultrathin-film electrodes with controlled architectures for enhanced energy storage and long-term cycling stability.

## 2 Materials and Methods

### 2.1 Synthesis of POSS Oligomers

POSS oligomers were synthesized as mixtures of oligomeric silsesquioxanes with a polyhedral structure and their analogs with open chains. Detailed synthesis steps and chemical characterization are included in prior publication and illustrated in Figure S1.^19^ POSS oligomers were dispersed in ethanol at a concentration of 0.05 mg/mL for experiments in this work.

### 2.2 Synthesis of MXene

Ti_3_C_2_T_*x*_ (Figure S2) was synthesized as previously reported, and highly concentrated (9 mg/mL) dispersions of MXene flakes in DI water were obtained (see the Supporting Information for more details).^21^ The dispersions were then diluted with DI water:ethanol 1:5 (v:v) to a concentration of 0.05 mg/mL.

### 2.3 Langmuir–Blodgett (LB) Films

A KSV 2000 minitrough was used to obtain the Langmuir isotherms and the LB films on piranha-treated silicon wafers and indium tin oxide (ITO) glass (thickness: 1.1 mm).^22,23^ MXene dispersions at a concentration of 0.05 mg/mL were spread on the aqueous subphase dropwise and left undisturbed to allow ethanol to slowly evaporate for 30 min. Equal amounts of solutions of the POSS oligomers in ethanol at a concentration of 0.05 mg/mL were then added similarly. Once all of the solvent was evaporated, compression isotherms were obtained at a rate of 5 mm/min. LB monolayered films were obtained by vertical dipping of the substrates at a rate of 1 mm/min at selected surface pressures. Multilayered LB films were obtained by repeated vertical dipping on a single substrate with a 30 min waiting time between each deposition cycle at a surface pressure of 15 mN/m. This surface pressure was selected because it led to good MXene surface coverage with no aggregations, allowing for a higher electrolyte accessible surface area.

### 2.4 Fabrication of Supercapacitors

Two-electrode symmetric supercapacitors were fabricated using two of the LB multilayered films coated on ITO glass and a poly(vinyl alcohol) (PVA)/H_2_SO_4_ gel electrolyte. The gel electrolyte was prepared by adding 1 g of PVA in 10 mL of deionized water.^24^ The mixture was heated at 80 °C for 1 h under stirring.^24^ Then, the mixture was left to cool down to room temperature and 0.5 mL of H_2_SO_4_ was added.^24^ The gel electrolyte was spread between the two LB electrodes, and polyimide tape (Bertech) was placed around the supercapacitor to provide external insulation. The total thickness of the supercapacitor was ∼2.5 mm, the electrolyte thickness was ∼0.3 mm, the ITO substrate thickness was 1.1 mm, and the MXene/POSS thickness varied from 100 to 400 nm depending on the number of layers and the POSS peripheral composition, as discussed in more detail in later sections. The active electrode area was 2.5 cm^2^.

### 2.5 Characterization

Zeta potential and dynamic light scattering (DLS) measurements of MXenes, POSS oligomers, and their mixtures in water (0.1 mg/mL) were conducted using a Zetasizer Nano ZS (Marven Instruments) at 633 nm and a scattering angle of 173°. Polystyrene cuvettes were used, and an average of three measurements was obtained.

UV–vis spectroscopy measurements were conducted using a Shimadzu UV-3600 Plus Spectrophotometer within 300–800 nm.

Contact angle measurements were performed by drop-casting 40 μL of water droplets onto the multilayered films. Images were taken within the first 10 s of the water droplet application using a KSV CAM101 system.

Scanning electron microscopy (SEM) images were obtained on a Hitachi SU-8230 electron microscope operated at 3 kV voltage without sputtering. For the cross-sectional images, the samples on ITO glass were attached perpendicular to the SEM mount. Samples were sputter-coated with gold for 45 s at 30 mA (the resulting gold layer thickness is ∼3 nm). EDX was performed using a Hitachi-3400SN SEM instrument with Oxford EDX on unsputtered films.

AFM was conducted by using a Bruker Dimension Icon microscope. AFM images were collected at tapping mode in air using AFM probes (HQ:XSC11/AI BS) with a tip radius of 8 nm and a spring constant of 3–16 N/m according to the usual procedure.^25^ High-resolution scans were collected using ultrasharp AFM probes (MikroMasch, Hi’Res-C18/Cr–Au) with 1 nm tip radius and 2.8 N/m spring constant. All scans were collected with a scan rate of 0.5 Hz and a resolution of 512 × 512.

X-ray photoelectron spectroscopy (XPS) measurements of the multilayered films were conducted using a Thermo K-Alpha XPS instrument from Thermo Scientific with Al Kα (*hm* = 1476 eV) radiation. Survey scans (average of two scans) were collected with a dwell time of 50 ms and a step size of 1.0 eV. High-resolution scans (average of 10 scans) were collected with a dwell time of 50 ms and a step size of 0.1 eV. The C 1s peak at 284.5 eV for sp^2^-hybridized carbon atoms was used to calibrate all spectra. Background corrections (Shirley) and Gaussian–Lorentzian (GL) peak shape fitting were performed using CasaXPS. The fwhm and position of the peaks were constrained to perform the deconvolution of the high-resolution C 1s and Ti 2p peaks, and the Ti 2p_1/2_ and 2p_3/2_ components were constrained so that the area ratio would be 2:1.^26,27^

X-ray diffraction (XRD) measurements were performed using a Rigaku SmartLab SE with Co Kα radiation and Bragg–Brentano beam (BB) optics. Measurements were conducted with a step size of 0.01 and a rate of 10°/min.

Electrochemical testing was conducted by using VersaSTAT3 and Zahner Zennium potentiostats. All supercapacitors were preconditioned at 20 mV/s for 10 cycles. Cyclic voltammetry was conducted at variant scan rates from 5 to 100 mV/s within a potential window of 0–0.6 V. The specific capacitance was calculated using the equation *C* = 2· , where *V* is the voltage, *I* is the current, Δ*V* is the potential window, ν is the scan rate, and *a* is the area of the two electrodes.^3,28^ Electric conductivity was measured using an Agilent E5272A source/monitor unit.

Prolonged retention experiments up to 10,000 cycles were conducted at a scan rate of 20 mV/s. Charge–discharge experiments were conducted at variant current densities of 0.02–0.1 mA/cm^2^ with a potential window of 0–0.6 V, and the specific capacitance was calculated from the equation *C* = 4·, where Δ*t* is the discharge time. Finally, the specific energy was calculated using the equation *E* = ·*C*·Δ*V*^2^.^3^ Electrochemical impedance spectroscopy (EIS) measurements were conducted at the open circuit potential (OCP) with 10 AC mV amplitude in a frequency range of 1 MHz to 10 mHz.

## 3 Results

**Figure 1:**
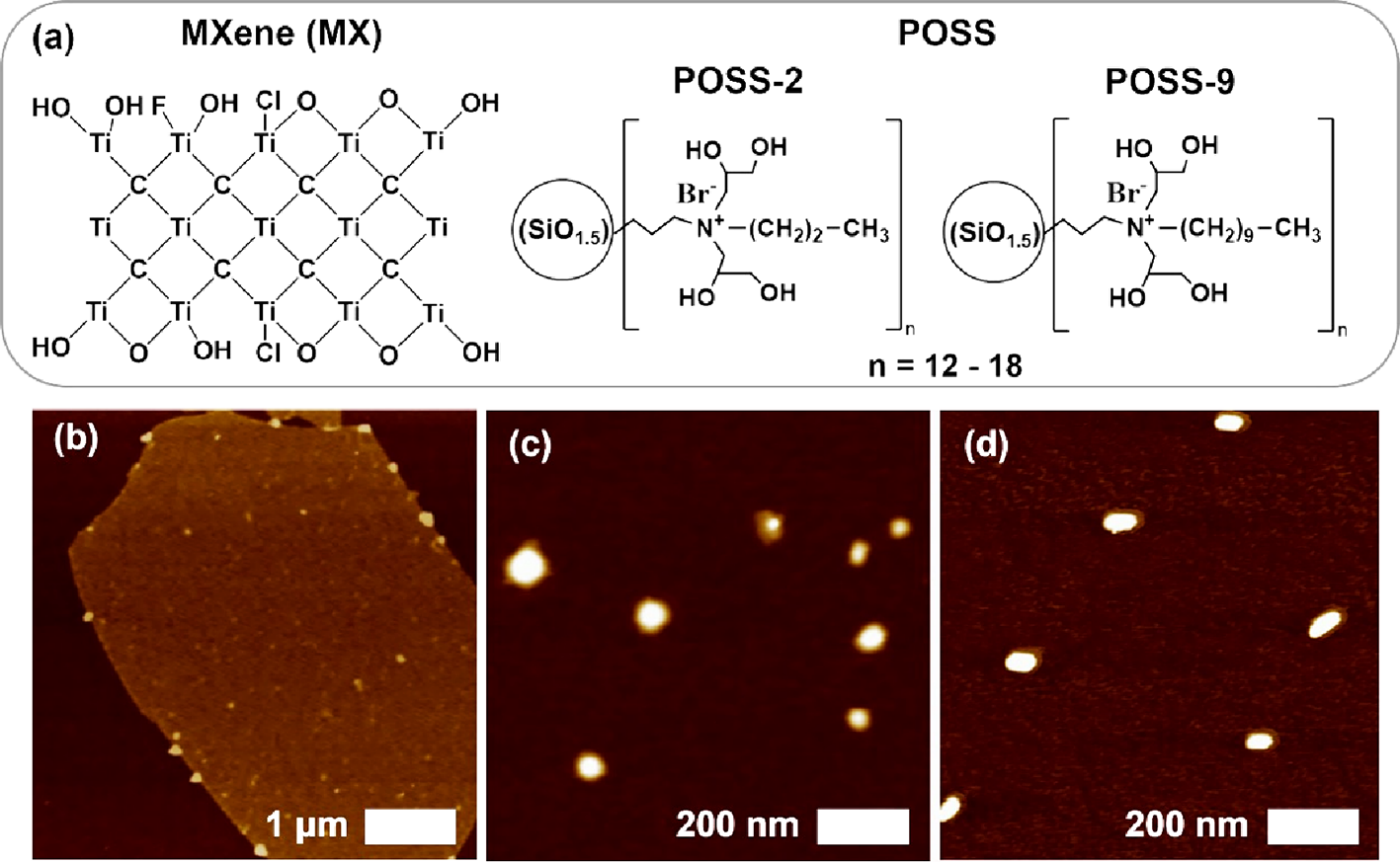
(a) Chemical structures for Ti_3_C_2_T_*x*_ MXene and POSS-2 and POSS-9 nanoparticles. High-resolution AFM topography images for (b) MXene, (c) POSS-2, and (d) POSS-9. The *Z*-axis is 15 nm for all of the AFM images.

AFM topography images of drop-cast aqueous dispersions show MXene (MX) nanosheets with a thickness of 1.7 ± 0.4 nm, a length of 4.8 ± 1.2 nm, and a width of 2.5 ± 0.9 nm, similar to previous reports on Ti_3_C_2_T_*x*_ MXenes (Figure 1).^29,30^ Synthesized POSS nanoparticles have diameters of 2.7 nm with narrow size distribution.^19^

After adsorption, the functionalized POSS with short alkyl chains (POSS-2) formed flat disklike micelles with diameters of 69.7 ± 9.7 nm and heights of 6.8 ± 2.0 nm, while POSS with longer alkyl chains (POSS-9) formed flat cylindrical micelles with lengths of 115.6 ± 14.9 nm, widths of 73.6 ± 6.8 nm, and heights of 9.8 ± 2.2 nm (Figure 1 and Figure S3).

Overall, the MXene nanosheet component in aqueous dispersions had hydrodynamic diameters of 1600 ± 390 nm and ζ-potential values of −27 ± 1 mV (Figure S4 and Table S1).^26,31^ On the corresponding nanoparticle suspension, POSS-2 had hydrodynamic diameters of 72 ± 6 nm with ζ-potential values of 30 ± 2 mV and POSS-9 exhibited diameters of 140 ± 40 nm with ζ-potential values of 41 ± 2 mV.

These values are comparable to other amphiphilic functionalized POSS reported in the literature.^32−34^ The size and ζ-potential values reveal the effect of the POSS alkyl chain length on their assembly in aqueous solutions due to the varying hydrophobic/hydrophilic balance (Figure 1a). Specifically, the larger size of POSS-9 is attributed to the enhanced hydrophobicity of the longer alkyl chains that leads to the formation of larger aggregates in aqueous dispersions.^19^ Furthermore, the positive ζ-potential values are attributed to the dominated positively charged quaternary ammonium surface terminal groups, while the lower values for POSS-2 indicate partial charge screening of the ammonium cations.^19^

The addition of POSS to the MXene dispersions caused decreased hydrodynamic diameter values of 1320 ± 40 and 1290 ± 190 nm for MX/POSS-2 and MX/POSS-9, respectively, due to the introduction of the much smaller POSS-2 and POSS-9 nanoparticles (Figure S4b and Table S1). Furthermore, ζ-potentials shifted to 10 ± 1 and 19 ± 1 mV for MX/POSS-2 and MX/POSS-9, respectively. The dramatic changes in ζ-potential values are indicative of dominating surface charge screening of the MXene surface groups, resulting from the adsorption of the POSS nanoparticles on the MXene nanosheet surfaces.^35^

### 3.1 Monolayer Assembly

**Figure 2:**
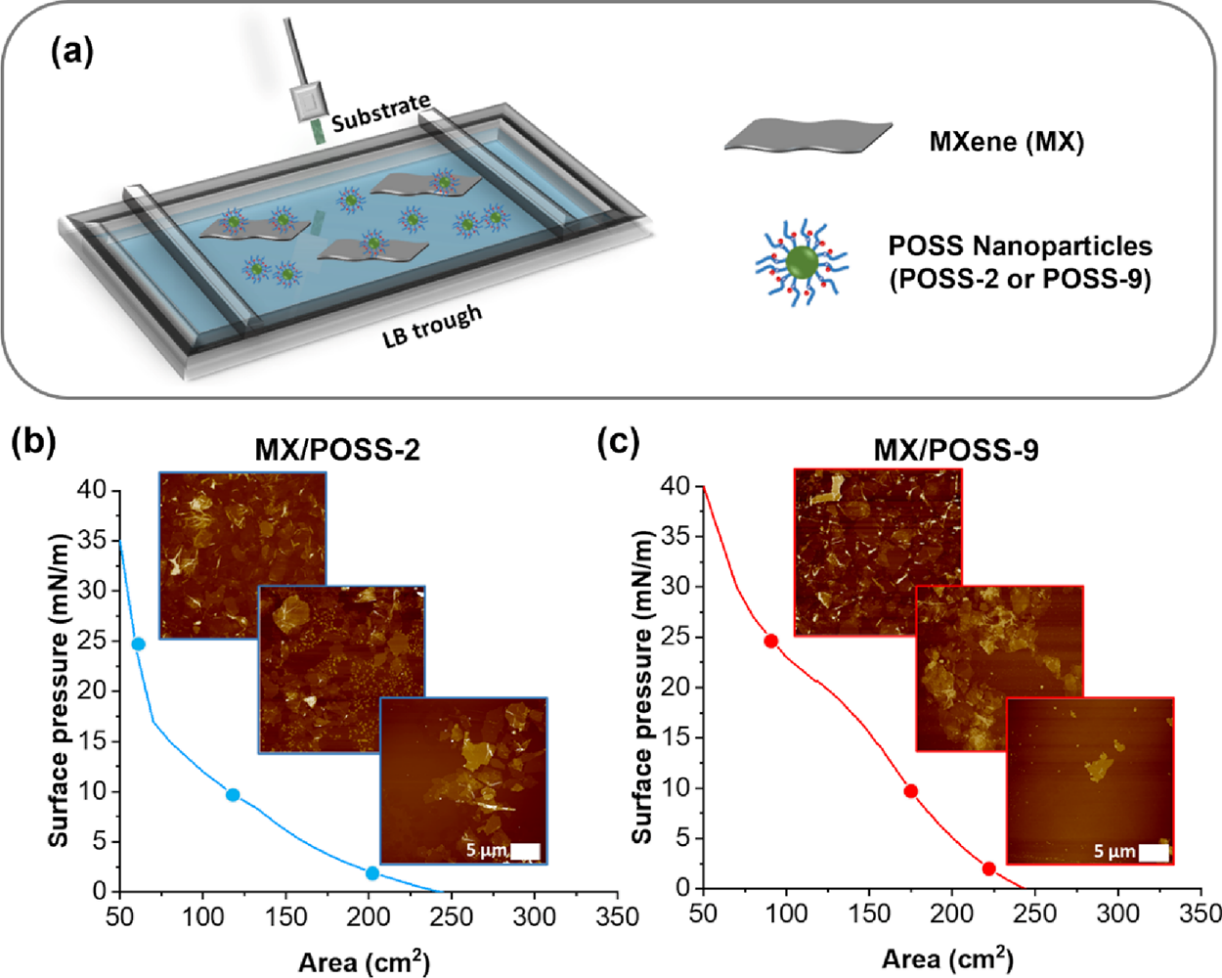
(a) Schematic representation of mixed Langmuir monolayer formation. Langmuir isotherms for (b) MX/POSS-2 and (c) MX/POSS-9. Insets show AFM topography images. The same scale bar of 5 μm applies to all AFM images. The *Z*-axis is 160 nm for MX/POSS-2, 25 mN/m, 15 nm for and MX/POSS-9, 2 mN/m, and 60 nm for all other images.

As is known, MXene particles form stable colloids due to their inherent hydrophilicity and negative surface charge. As a result, it was not possible to create monolayers at the air–water interphase as discussed in other studies.^36^ Special approaches were suggested to create stable monolayer MXene films at the liquid–air interface.^37^ It is important to note that, in contrast to many other reports,^17^ the mixed MX/POSS materials in this study formed stable Langmuir monolayers at the water–air interphase due to the amphiphilicity of the POSS nanoparticles (Figure 2a). In this mixed state, MXenes and POSS hydroxyl groups submerged in the aqueous phase, while the POSS hydrophobic silsesquioxane cores and alkyl chains prevent their total submersion in water.

Figure 2b,c shows the surface pressure–area isotherms for the MX/POSS-2 and MX/POSS-9 prepared in this study, which exhibited shapes similar to other amphiphilic materials.^38,39^ Three main characteristic areas are apparent and correspond to the traditional gas, liquid, and solid phases.^40^ The isotherms for MX/POSS-9 shifted to larger surface areas, indicating the formation of less compact morphologies.^40^ The increased length of the POSS-9 hydrophobic alkyl chains may prevent the MXene nanosheets to closely pack at the air–water interphase, increasing the effective surface area they occupy and leading to loosely condensed morphologies.^41^ Surface-pressure isotherms for pure POSS-2 and POSS-9 follow the same behavior (Figure S5).

Upon decompression, the Langmuir isotherms for MX/POSS-2 and MX/POSS-9 show large hysteresis that can be attributed to the strong interfacial interactions between the positively charged ammonium terminal groups of the POSS particles and the negatively charged surface groups of the MXene flakes as well as to hydrogen bonding interactions between the nanoparticles and the flakes (Figure S6). As shown in Figure S7, these interactions do not allow the full recovery of the initial morphologies, resulting in compacted morphologies during the second compression cycle that is, in contrast, completely reversible.^40,41^

**Figure 3:**
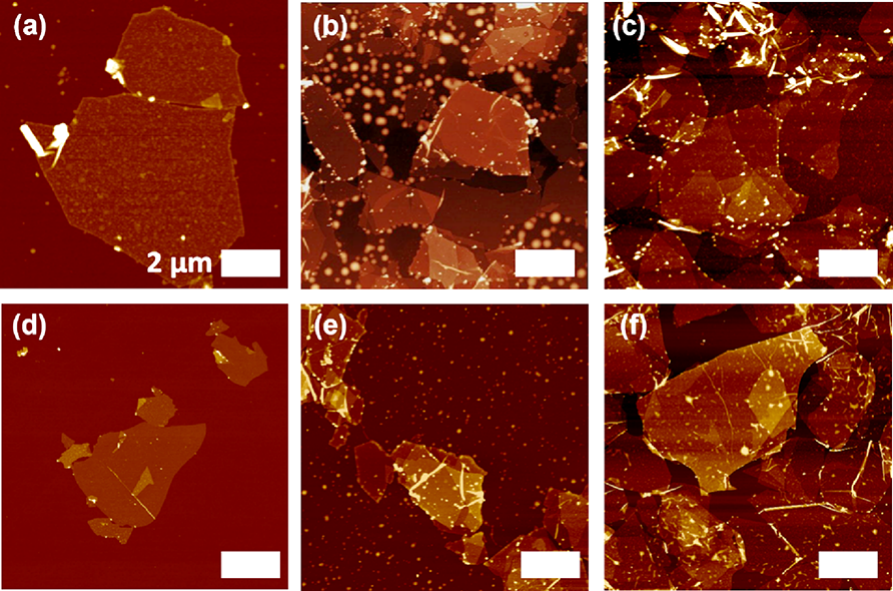
AFM topography images for (a–c) MX/POSS-2 and (d–f) MX/POSS-9 at (a, d) 2 mN/m, (b, e) 10 mN/m, and (c, f) 25 mN/m. All panels have a scale bar of 2 μm and a *Z*-axis of 30 nm.

Morphological changes during compression were observed for POSSs with different alkyl chain peripheral compositions (Figure 3 and Figure S8). Specifically, at a pressure of 2 mN/m, single MXene nanosheets with POSS micelles of 5.0 ± 0.5 nm in height and 200 ± 12 nm in diameter spread between the MXenes were observed for MX/POSS-2. At the same pressure, only MXene nanosheets were visible for the MX/POSS-9 monolayers. Higher surface pressures led to more compact morphologies in both cases. Specifically, at 10 mN/m, MX/POSS-2 showed aggregated MXene nanosheets with larger POSS micelles of 17 ± 2 nm in height and 400 ± 50 nm in diameter spreading between the MXene nanosheets.

The increased size of the POSS micelles at a higher surface pressure indicates their aggregation. Surprisingly, the larger POSS alkyl chains of MX/POSS-9 led to smaller micellar morphologies with heights of 9 ± 3 nm. The formation of larger micelles for POSS-2 can be related to the strong hydrogen bonding and ionic interactions associated with the hydroxyl end groups and the positively charged ammonium terminal groups and bromine counterions on the surface of the silsesquioxane nanoparticle.^17^ On the other hand, longer alkyl chains prevent aggregation at the air–water interphase due to steric hindrance effects.^42^ Upon compression, the MXene nanosheets form larger aggregates, while the POSS micelles are trapped between the MXene stacks.

### 3.2 Formation of Multilayered Films

**Figure 4:**
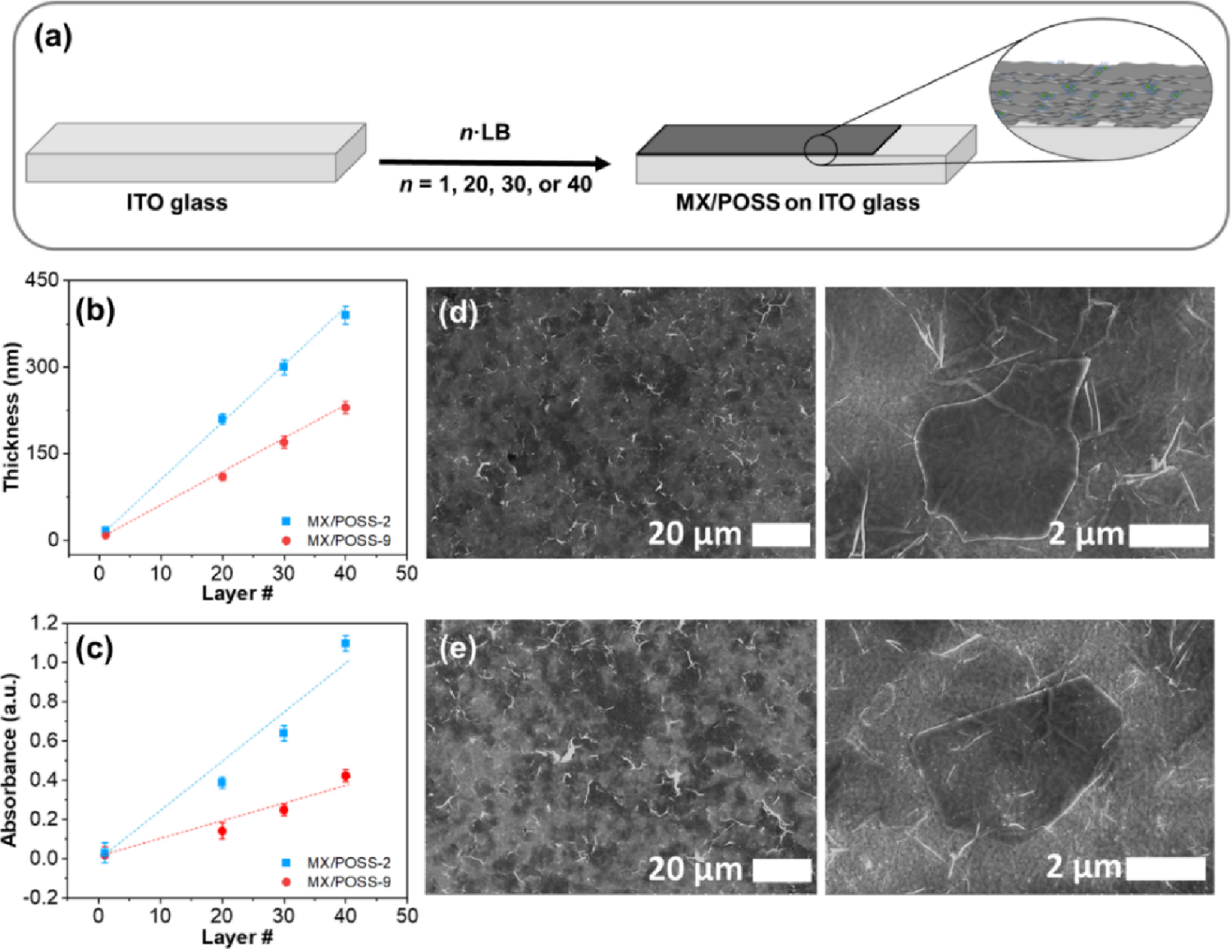
(a) Schematic representation of the fabrication of multilayer films. (b) Thickness and (c) absorbance at 770 nm vs the number of layers. (d, e) SEM surface images for (d) MX/POSS-2 and (e) MX/POSS-9 composed of 40 layers.

Multilayered films showed consistent linear growth with an increase in the number of Langmuir monolayers deposited (Figure 4a,b). Specifically, the thickness of the MX/POSS-2 films linearly increased to 390 ± 15 nm with an increase in the number of monolayers to 40 (Figure 4b).

These observations were further confirmed using UV–vis spectroscopy, where a linear increase in absorbance with the number of layers and higher absorbance values for the MX/POSS-9 films were also observed (Figure 4c and Figure S9).

The calculated effective thickness of the individual layer within multilayered films was calculated to be 9.7 nm, which is lower than the thicknesses (∼17 nm) measured for the first layers formed under the same conditions. Similarly, the thickness of the MX/POSS-9 multilayered films increased to 230 ± 11 nm as the number of layers increased to 40, which corresponds to the effective thickness of 5.6 nm per layer, which is much lower than 9 nm for the first monolayer.

We suggest that the differences in the layer thickness in the multilayers as compared to their first monolayer initially deposited on silicon indicate that the initial gaps between the nanosheets and nanoparticles are intermixed during sequential deposition, leading to higher connectivity within and across adjacent layers and, thus, reduced effective thickness of further mixed layers. According to AFM images, the POSS nanoparticles are mostly located at the air gaps between the MXene stacks and very few of them adsorbed on the flake surface. Furthermore, no clear evidence of intercalation in between individual MXene nanosheets in multilayered films was found with XRD (Figure S10). Indeed, *d*-spacing values of 0.84–0.88 nm were calculated by the very broad peak at 11.5–12.3°, which corresponds to the (002) plane of MXenes.^43^ These values are comparable to the *d*-spacings reported in the literature for pure MXenes (∼ 0.9 nm), thus confirming no expansion of intersheet spacing in the presence of POSS nanoparticles.

Finally, the uniform surface coverage of the multilayers with interconnected nanosheets and micelles was confirmed using SEM (Figure 4d,e and Figures S11 and S12). Furthermore, the higher thickness of the MX/POSS-2 films as compared to the MX/POSS-9 films can be attributed to the larger sizes of the aggregated POSS-2 micelles prepared under the same surface pressure. The aggregated micelles and their presence in intersheet areas increase the effective thickness of the mixed monolayers, subsequently increasing the total effective thickness of the multilayered films without significant intercalation.

**Figure 5:**
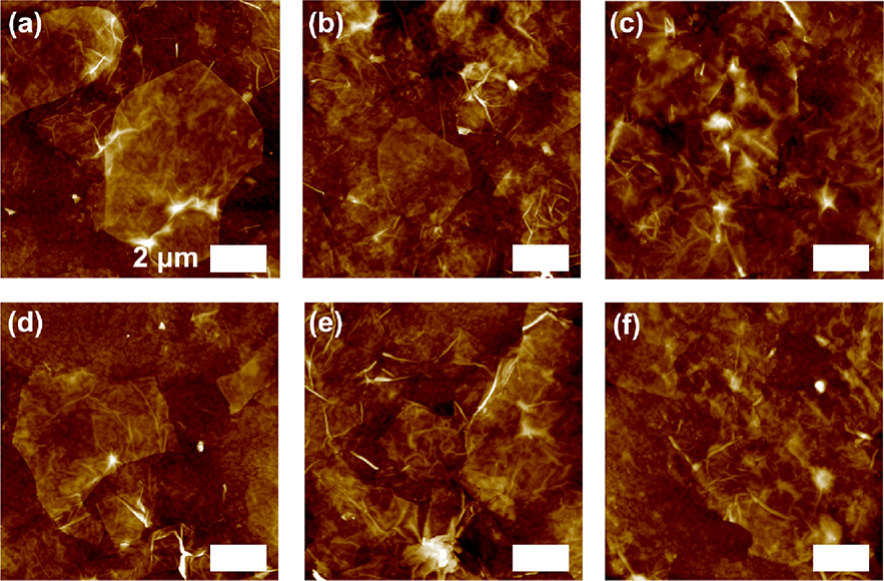
AFM topography images for (a–c) MX/POSS-2 and (d–f) MX/POSS-9 multilayers of (a, d) 20 layers, (b, e) 30 layers, and (c, f) 40 layers. All panels have a scale bar of 2 μm and a *Z*-axis of 140 nm.

AFM topography images revealed the presence of individual large MXene flakes on the surface of multilayers composed of 20 and 30 layers (Figure 5 and Figure S13). Furthermore, increasing the number of layers led to rougher surfaces composed of wrinkled MXene flakes. Indeed, the RMS roughness increases from 1.2 ± 0.3 nm (individual monolayer) to 13.8 ± 2.1 nm (for 40 layered films) for the MXene-covered areas (1 × 1 μm surface areas). On the other hand, the changes in the peripheral composition of POSS nanoparticles did not lead to significant changes in the surface morphology of the different multilayered films. We suggest that POSS micelles remain trapped between the deposited layers and do not significantly alter the final surface morphology which is dominated by the densely packed MXene nanosheets.

**Figure 6:**
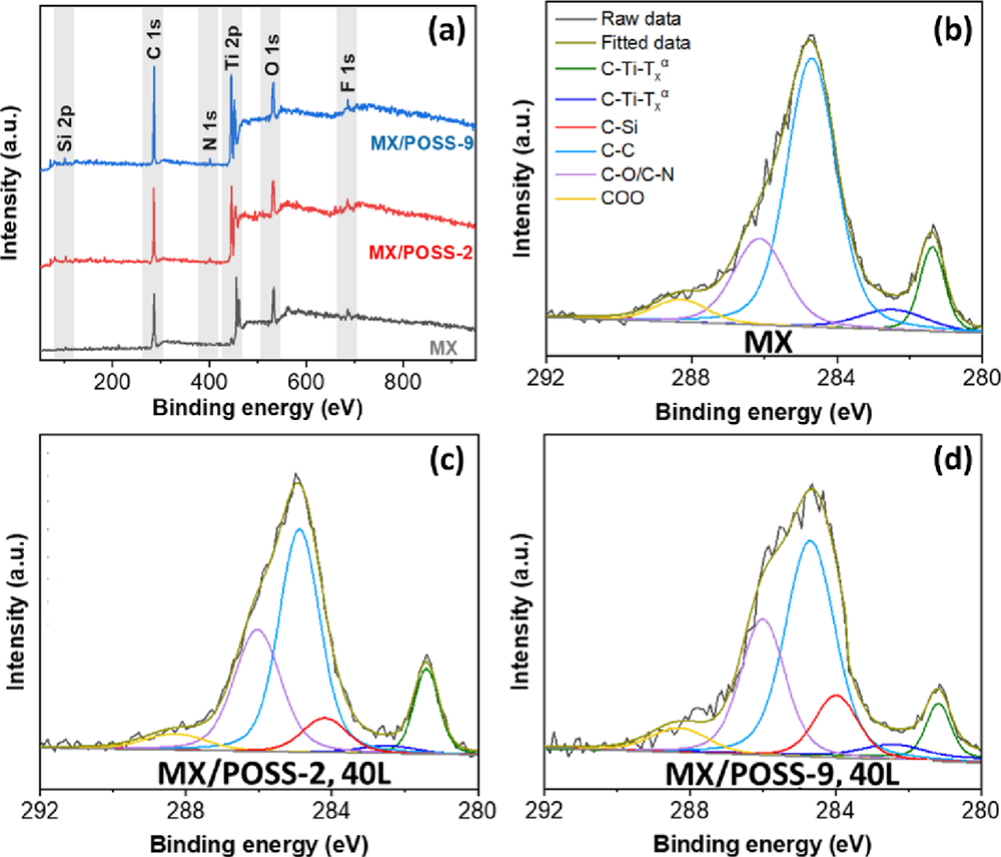
XPS (a) survey scan and high-resolution C 1s deconvoluted peaks for (b) pure MX, (c) MX/POS-2, 40L, and (d) MX/POSS-9, 40L films. The legend in panel (b) applies also to panels (c) and (d).

The presence of buried POSS nanoparticles underneath the MXene nanosheets was verified by using XPS (Figure 6a and Table S2). XPS survey scans of pure drop-cast MXene films revealed a composition of 51.9 at % carbon, 20.9 at % oxygen, 18.9 at % titanium, and small amounts (<7 at %) of fluorine and chlorine, which is similar to other reports on MXenes.^27,29,30^ The hybrid multilayered films exhibited additional peaks attributed to nitrogen (1.1–1.9 at %), silicon (5.9–6.2 at %), and bromine (1.1–1.7 at %) resulting from the silsesquioxane cores, tertiary nitrogen atoms, and bromine counterions of the modified POSS nanoparticles within multilayered films. Furthermore, all films showed strong C–Ti-T_*x*_ (6.6–12%) resulting from the MXene nanosheets. Deconvoluted C 1s peaks further verified the presence of the buried POSS nanoparticles (Figure 6b–d and Table S3). The presence of POSS was further verified by EDS Si mapping of the surface of the composite multilayered films (Figure S14).

Indeed, the composite multilayered films showed C–Si peaks (7.7–10.2%) from the POSS cores in addition to C–C (42.9–56%), C–O/C-N (20.1–28.5%), and COO (5.0–5.3%) bonds associated with the MXenes and/or POSS nanoparticles for all films. High-resolution Ti 2p peaks (Figure S15 and Table S4) showed no significant changes with POSS nanoparticle addition, indicating that the MXene nanosheets remained intact in the mixed layer.

### 3.3 Electrochemical Performance

**Figure 7:**
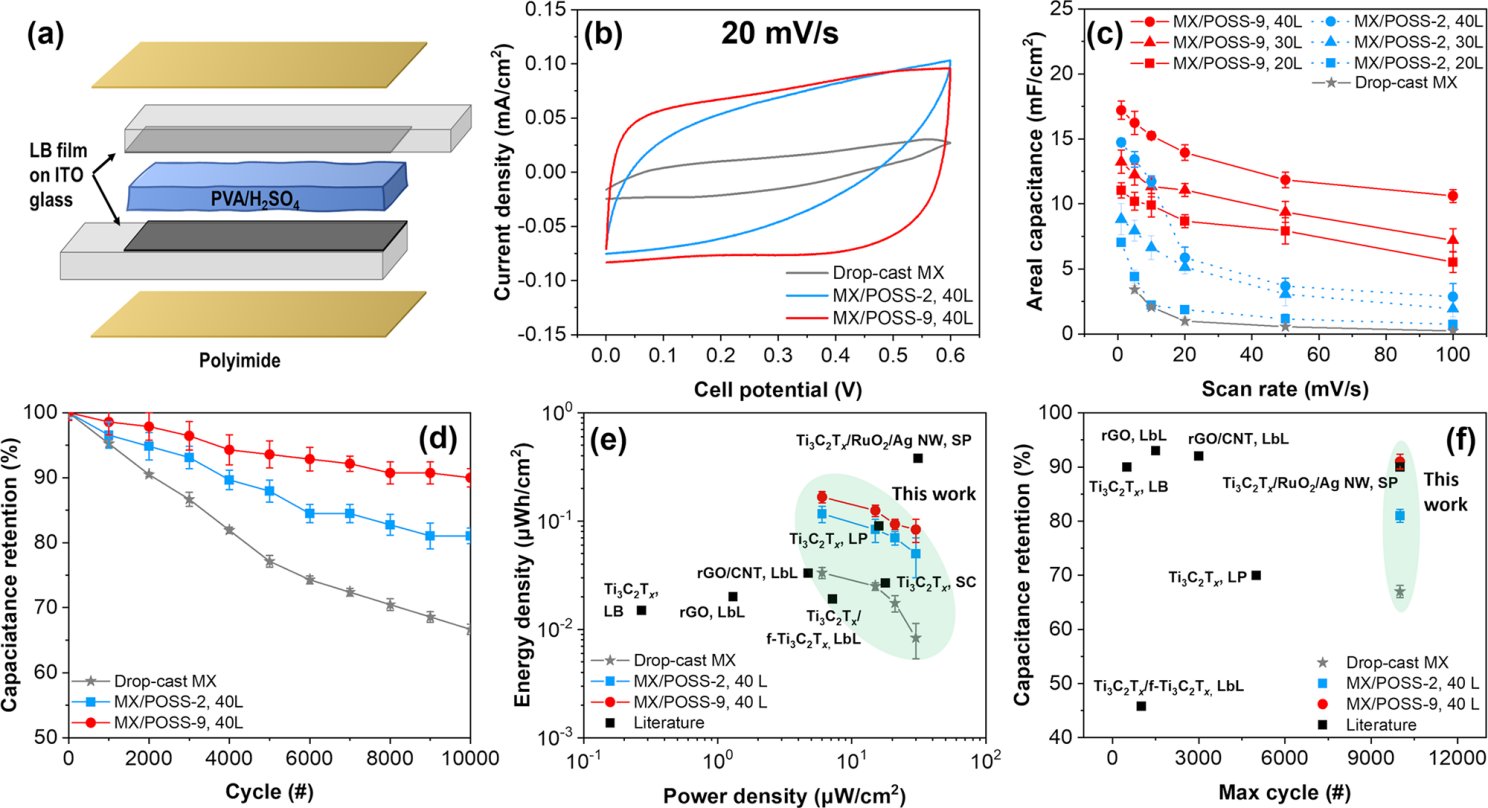
(a) Schematic representation of a two-electrode solid-state supercapacitor. (b) Cyclic voltammograms for drop-cast MX, MX/POSS-2, 40 L, and MX/POSS-9, 40 L, at 20 mV/s. (c) Areal capacitance vs scan rate for all MX, MX/POSS-2, and MX/POSS-9 electrodes. (d) Capacitance retention vs cycle number for drop-cast MX, MX/POSS-2, 40 L, and MX/POSS-9, 40 L, at 20 mV/s. (e) Energy density (μWh/cm^2^) vs power density (μW/cm^2^) Ragone plot and (f) capacitance retention vs max cycle number.

As shown in Figure 7a and Figure S16, to investigate the effect of POSS addition and POSS peripheral composition variation on the energy storage properties of the MX/POSS multilayers, two-electrode symmetric supercapacitors were fabricated similarly to the literature.^6,44^

First, all MX/POSS supercapacitors fabricated here exhibited pseudorectangular cyclic voltammograms (CVs), indicative of the electric double formation (Figure 7b–d and Figures S17 and S18).^45,46^ On the other hand, drop-cast MX with similar thickness values (300 nm) showed highly polarized CVs, indicating ion-diffusion limitations possibly resulting from the highly aggregated, compacted MXene assemblies without a preferred orientation.^47,48^ Drop-cast MX films exhibited areal capacitance values of 3.5 ± 0.3 mF/cm^2^ at 1 mV/s, comparable to previous reports using gel electrolytes.^49−51^

It is important to note that the addition of POSS nanoparticles led to significantly higher areal capacitance values despite the increased hydrophobicity and the decreased electric conductivity from 46 ± 3 S/cm for drop-cast MX to 35 ± 1 S/cm for MX/POSS-2, 40L, and 27 ± 1 S/cm for MX/POSS-9, 40L, due to the dilution of the conductive MXene nanosheets (Figure 7c and Figure S19). Specifically, MX/POSS-2 films of 20 layers had capacitance values of 7.1 ± 0.5 mF/cm^2^ and MX/POSS-9 films of 20 layers had capacitance values of 11.0 ± 0.6 mF/cm^2^ at 1 mV/s. Increasing the number of layers leads to higher capacitance values due to the increase in the electrochemically active material content.^46^

Moreover, MXene/POSS electrodes exhibited a much higher long-term stability under cyclical loading (Figure 7d). This much higher stability suggests that the addition of POSS nanoparticles prevents MXene flake restacking, facilitating ion transport and higher available surface areas, and as a result, it improves energy storage performance.^52^ This suggestion was further confirmed by a decrease in the charge transfer resistance (*R*_tc_) from 1.2 kΩ for drop-cast MX to 0.9 kΩ for MX/POSS-2, 40L, and 0.5 kΩ for MX/POSS-9, 40L (Figure S20).

Next, the energy and power density were calculated from galvanostatic charge–discharge experiments, and they are summarized in a Ragone plot for drop-cast MX, MX/POSS-2, 40L, and MX/POSS-9, 40L, films (Figure S21 and Figure 7e). As we observed, drop-cast MX demonstrated a maximum energy density of 0.03 μhW/cm^2^ and a power density of 30 μWh/cm^2^, which is comparable to the literature data.^53^ The addition of POSS-2 and POSS-9 nanoparticles led to 267 and 467% increases in energy density, respectively.

Furthermore, we compared these results against other MXene-based supercapacitors, including Ti_3_C_2_T_*x*_ (LB-deposited), Ti_3_C_2_T_*x*_ SC (spray-coated), Ti_3_C_2_T_*x*_ LP (laser-printed), Ti_3_C_2_T_*x*_/amine-functionalized (f-Ti_3_C_2_T_*x*_) LbL (layer-by-layer), Ti_3_C_2_T_*x*_/RuO_2_/silver nanowires (Ag NW) SP (screen-printed), and non-MXene-based, such as rGO LbL and rGO/carbon nanotube (CNT) LbL.^13,16,53−58^ Within this class, Ti_3_C_2_T_*x*_/RuO_2_/Ag NW exhibited the highest energy density value (0.38 μhW/cm^2^ at 31.3 μW/cm^2^). The high performance of Ti_3_C_2_T_*x*_/RuO_2_ is attributed to pseudocapacitive contributions arising from RuO_2_ and the highly conductive continuous network of Ag nanowires.^58^ Overall, from the analysis of these results, we can conclude that the hybrid MX/POSS-9, 40L, film shows second to the best energy density storage ability.

Finally, we observed improved cycling stability for the MX/POSS supercapacitor materials. For these measurements, prolonged cyclic voltammetry (up to 10,000 cycles) was conducted at 20 mV/s (Figure 7d). For these experiments, multilayered MX/POSS films with 40 layers were selected due to their superior energy storage performance. First, reference drop-cast MX films retained only 67% of their initial capacitance after a gradual decrease. The addition of POSS nanoparticles led to significant improvements in electrochemical stability, with MX/POSS-2, 40L, retaining 81% and, finally, the MX/POSS-9, 40L, film retaining 91% of their initial capacitance (as well as 95% after 5000 cycles). High-resolution XPS Ti 2p peaks did not show significant changes in the surface chemistry of the electrodes, thus indicating the absence of electrochemically triggered chemical reaction between components (Figure S22 and Table S5).

The enhanced cycling stability of the hybrid electrodes can also be attributed to the aggregation of POSS nanoparticles at the air gaps in between the stacked layers that prevent, to a great extent, further MXene restacking during prolonged cycling.^52^ Indeed, MXene-based electrodes reported to date frequently show irreversible capacitance loss, which usually leads to poor cycling stability with capacitance retentions of 46–90% after 500 to 5000 cycles.^4,13,16,55^ The irreversible capacitance loss is typically attributed to restacking of the MXene nanosheets, leading to insufficient electrolyte ion diffusion, as well as to MXene oxidative degradation, deteriorating electronic conductivity.^59^ As shown in Figure 7f and Table S6, in contrast, the improved cycling stability of the POSS-containing supercapacitors studied here is noteworthy in direct comparison with literature results on ultrathin-film (<500 nm electrode thickness) supercapacitors.^13,16,53,55−58^ Recent exceptions include reports on thick (>5 μm) shear-delaminated MXene electrodes with good cycling stability up to 500,000 cycles tested in a three-electrode configuration, while other strategies to achieve high capacitance retention include the development of three-dimensional electrodes.^60,61^

## 4 Conclusions

In conclusion, we designed and fabricated uniformly stacked hybrid MXene/POSS thin films with controlled thicknesses and high surface coverage at the air–water interface. Overall, the ultrathin-film hybrid POSS-containing MXene supercapacitor demonstrated greatly enhanced electrochemical stability compared to that of the corresponding pure MXene drop-cast films with comparable thickness.

The investigation of various compositions of POSS nanoparticles revealed their impact on the morphology of Langmuir monolayers and the corresponding multilayered films. Specifically, POSS nanoparticles with shorter alkyl chains formed larger aggregates between the MXene nanosheets due to hydrogen bonding and ionic interactions, while steric hindrance interactions were limited. The resulting electrodes exhibited enhanced power density and superior cycling stability in comparison with pure MXene drop-cast thin-film electrodes. These electrodes demonstrated up to 91% capacitance retention after 10,000 cycles. The enhanced energy storage performance can be attributed to the uniform deposition of MXene nanosheets at the air–water interface due to the mediation of the amphiphilic POSS micelles and the formation of porous channels within POSS micelles that facilitate ion transport. Furthermore, the presence of POSS micelles trapped in the open space between MXene nanostacks prevents further MXene restacking, thus improving the electrochemical stability during long-term cycling. This work provides an efficient route for the assembly and fabrication of hybrid thin-film electrodes utilizing 2D nanomaterials with enhanced ionic transport and energy storage capabilities. This approach may be equally useful when designing MXene electrodes for capacitive water deionization or developing sensors and ionotronic devices.

## Supporting Information Available

AFM topography and phase images, SEM images, electrochemical measurements, and additional characterizations (PDF)
