# Supplementary material for: Ultrathin Films of MXene Nanosheets Decorated by Ionic Branched Nanoparticles with Enhanced Energy Storage Stability
Source: ACS Appl Mater Interfaces. 2023 Nov 7;15(46):53776–85. doi: 10.1021/acsami.3c09064 (PMC10685356; doi:10.1021/acsami.3c09064)
Supplement: Supplementary file 1 — am3c09064_si_001.pdf [file am3c09064_si_001.pdf]

Supporting information for:

Ultrathin Films of MXene Nanosheets Decorated by Ionic Branched Nanoparticles with Enhanced Energy Storage Stability

Paraskevi Flouda,[a] Alex Inman,[b] Mariana Gumenna,[c] Daria Bukharina,[a] Valery V. Shevchenko,[c] Yury Gogotsi,[b] and Vladimir V. Tsukruk[a]*

[a]School of Materials Science and Engineering, Georgia Institute of Technology, Atlanta, Georgia 30332, USA

[b]A. J. Drexel Nanomaterials Institute and Department of Materials Science and Engineering, Drexel University, Philadelphia, Pennsylvania, 19104, USA

[c]Institute of Macromolecular Chemistry of the National Academy of Sciences of Ukraine, Kharkivske Shosse 48, Kyiv 02160, Ukraine

*E-mail: vladimir@mse.gatech.edu

Synthesis of POSS oligomers. A mixture of oligomeric silsesquioxanes with the general formula $[(HOCH_2CH(OH)CH_2)_2N(CH_2)_3SiO_{1.5}]_n$, where $(SiO_{1.5})_n$ is the inorganic core of the linear (a), branched (b), ladder (c), fully (d) or incompletely (e) condensed polyhedral structure was used as initial compound for the synthesis of nanoparticles containing ionic groups (**Figure S1**). The degree of polymerization *n* of oligomers in its composition ranged from 12 to 18.[1,2] Aprotic ionic groups containing quaternary ammonium cations with short propyl (compound POSS-2) or longer decyl (compound POSS-9) substituents were introduced into organic part of oligomeric silsesquioxanes by quaternization of tertiary nitrogen atoms of the initial compound with 1-bromopropane or 1-bromodecane at a ratio of N : Br = 1 : 1 (**Figure S1**).[3]

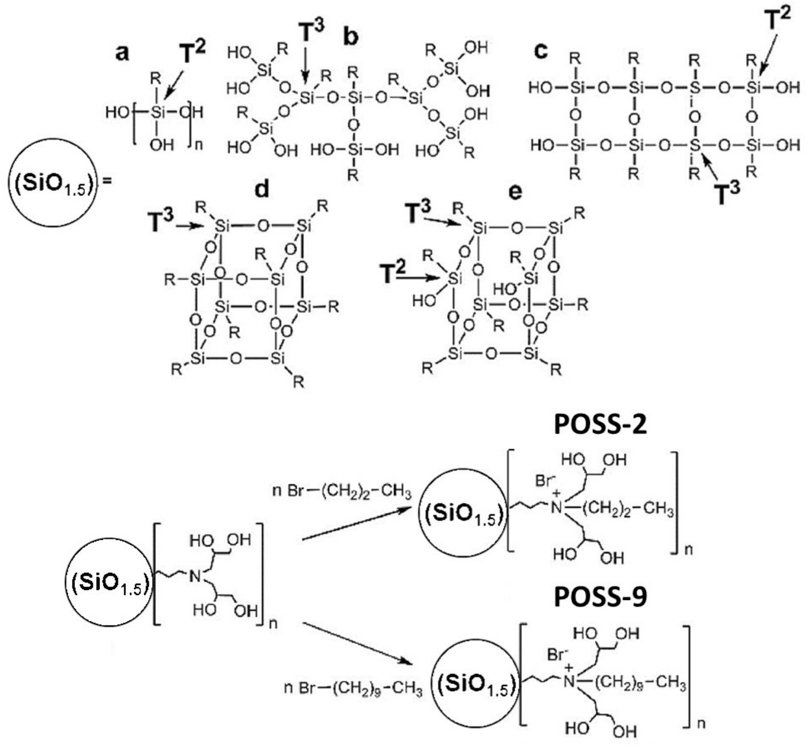

Figure S1. Schematic representation of the synthesis of POSS oligomers and chemical structures.[3]

Synthesis of MXene. The synthesis protocol followed as previously reported. $Ti_3C_2T_x$ MXene was synthesized by first making Ti_3AlC_2 MAX phase precursor.[4] Ti_3AlC_2 MAX was made by ball-milling TiC, Ti, and Al powders in a 2:1:1 mass ratio. It was then annealed at 1380 °C under an Ar atmosphere for 2 h. After annealing the MAX phase was washed with 9 M HCl overnight after which particles below 38 μm were separated. 5 g of the resulting MAX phase was then etched and

delaminated to form single flakes of MXene in a stable aqueous solution (**Figure S2**). To etch the MAX phase it was combined with 10 mL of HF (49%), 60 mL of HCl (36%), and 30 mL of DI water. The resulting was then stirred at 35 °C for 24 h. After neutralizing the solution to a pH > 6 the resulting multilayer $Ti_3C_2T_x$ was delaminated. To delaminate the etched multilayer $Ti_3C_2T_x$ 5 g of LiCl 250 mL of DI water was added and stirred for 24 h before finally being collected as delaminated $Ti_3C_2T_x$.

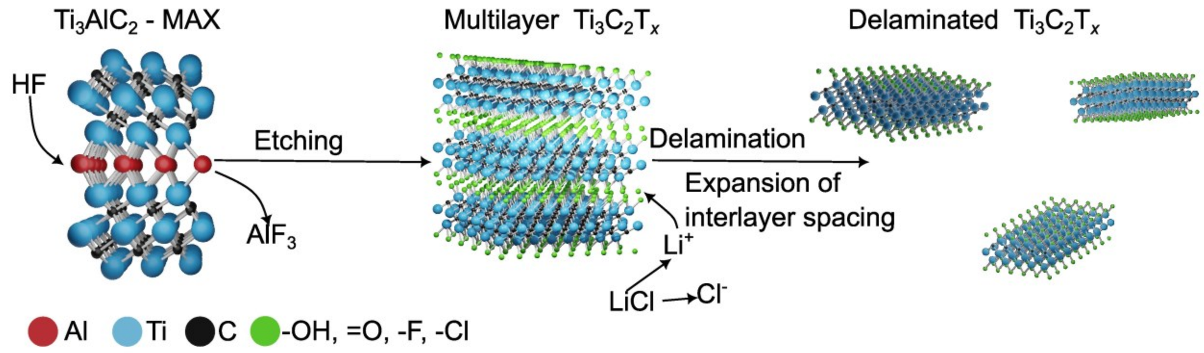

Figure S2. Schematic of the synthesis process for delaminated $Ti_3C_2T_x$. Beginning on the left, Ti_3AlC_2 is etched with HF resulting in multilayer $Ti_3C_2T_x$ (middle) which is then delaminated by expanding the interlayer spacing with the insertion of Li^+.

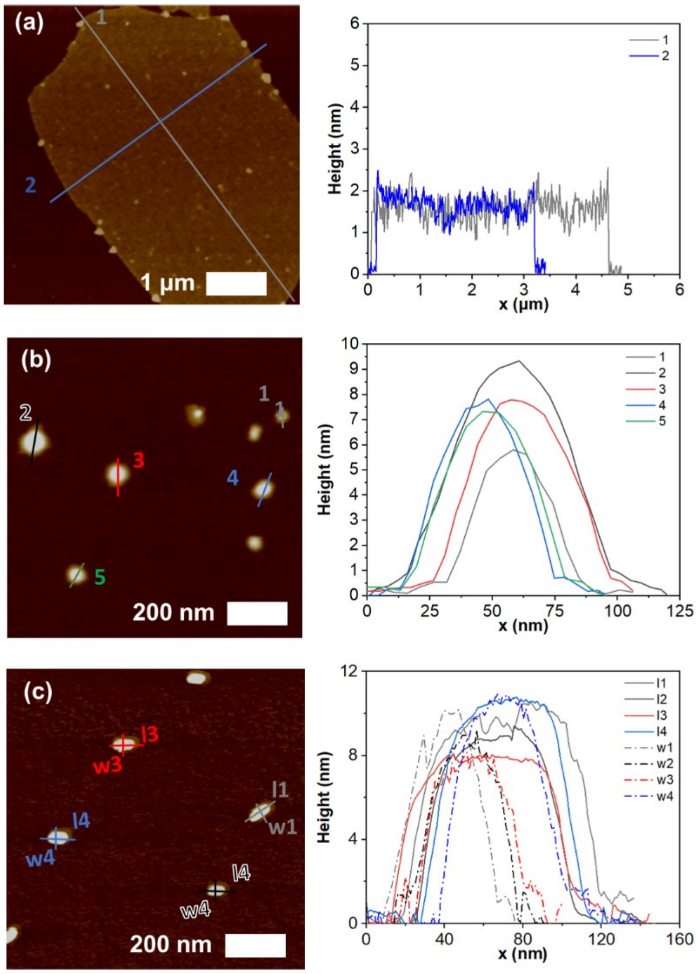

Figure S3. AFM topography images and height profiles for (a) MXene, (b) POSS-2, and (c) POSS-9. The Z-axis is 15 nm for all AFM images.

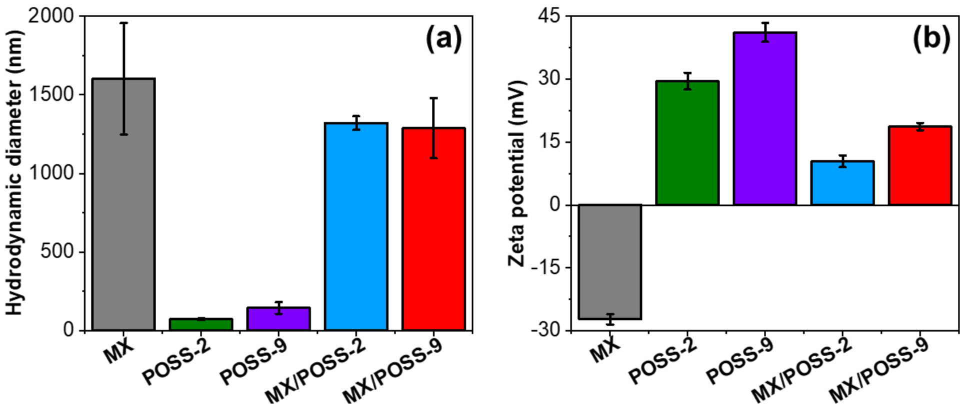

Figure S4. (a) Hydrodynamic diameters and (b) Zeta potential for MXene (MX), POSS nanoparticles (POSS-2 and POSS-9), and their mixtures (MX/POSS-2 and MX/POSS-9).

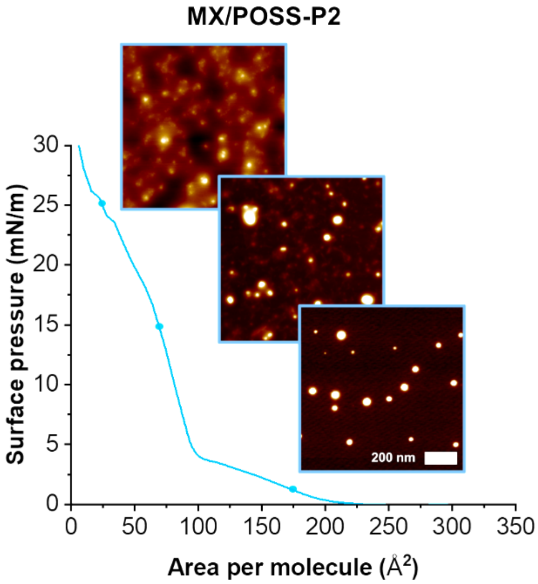

Figure S5. Langmuir isotherms for MX/POSS-2 and MX/POSS-9. Insets show AFM topography images. The z-axis is 15 nm for MX/POSS-2, 2 mN/m and MX/POSS-9, 2 mN/m, 15mN/m and 60 nm for all other images. The same scale bar of 200 nm applies to all AFM images.

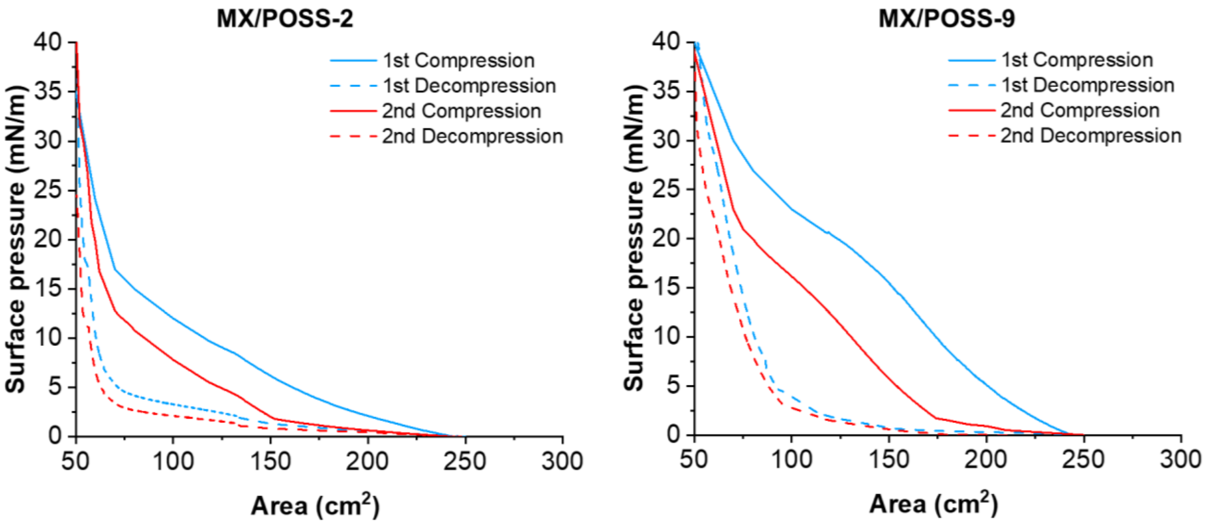

Figure S6. Langmuir isotherms for two compression-expansion cycles for MX/POSS-2 and MX/POSS-9 monolayers at the air-water interface.

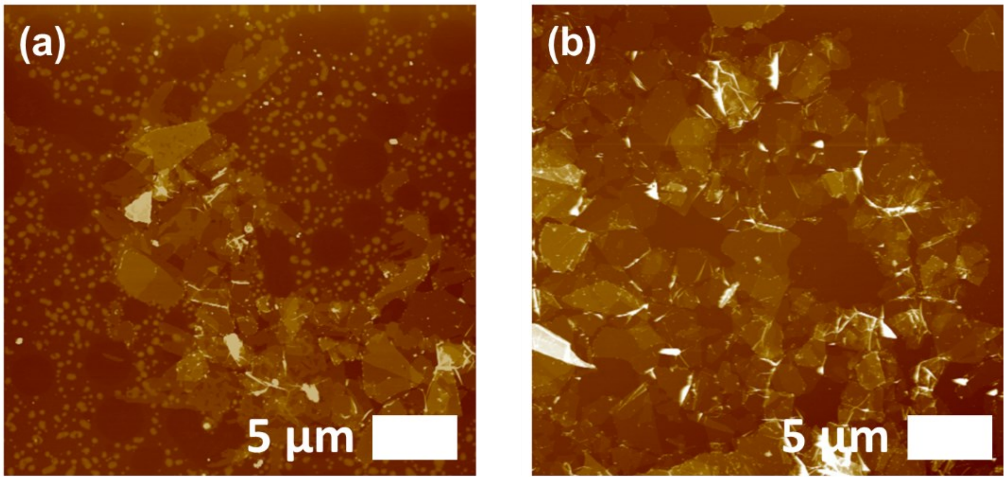

Figure S7. AFM topography images for second compression cycle at 15 mN/m for (a) MX/POSS-2 and (b) MX/POSS-9. The z-axis is 60 nm for both panels.

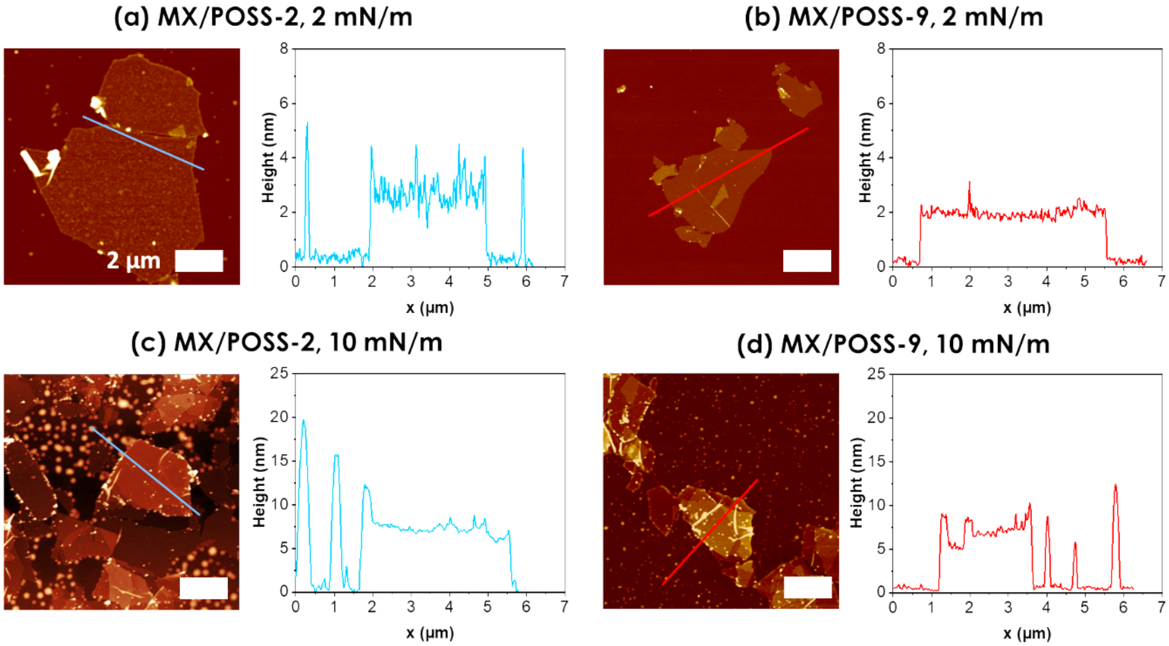

Figure S8. AFM topography images and corresponding height profiles for (a) MX/POSS-2 at 2 mN/m, (b) MX/POSS-9 at 2 mN/m, (c) MX/POSS-2 at 10 mN/m, and (d) MX/POSS-9 at 10 mN/m. All AFM images have a scale bar of 2 μm and a z-axis of 30 nm.

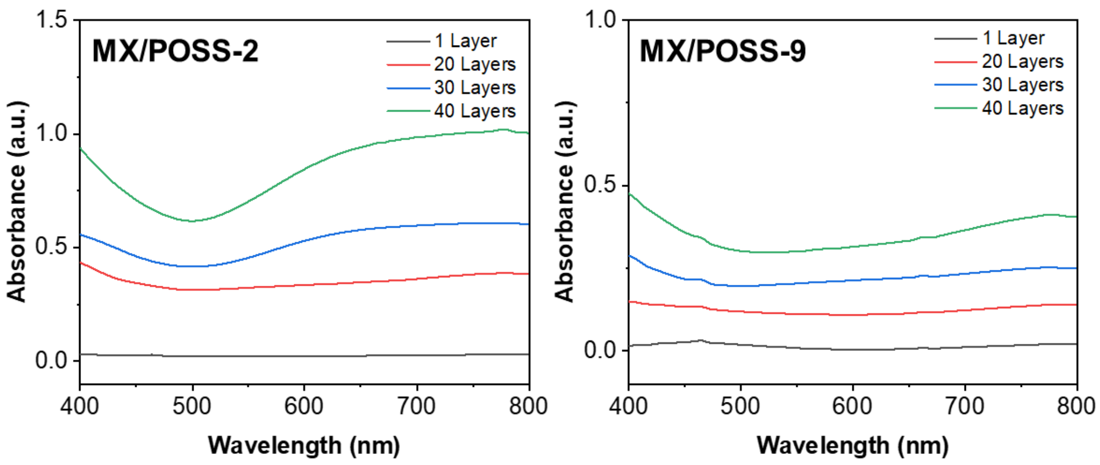

Figure S9. UV-Vis absorbance spectra for MX/POSS-2 and MX/POSS-9 for different numbers of layers.

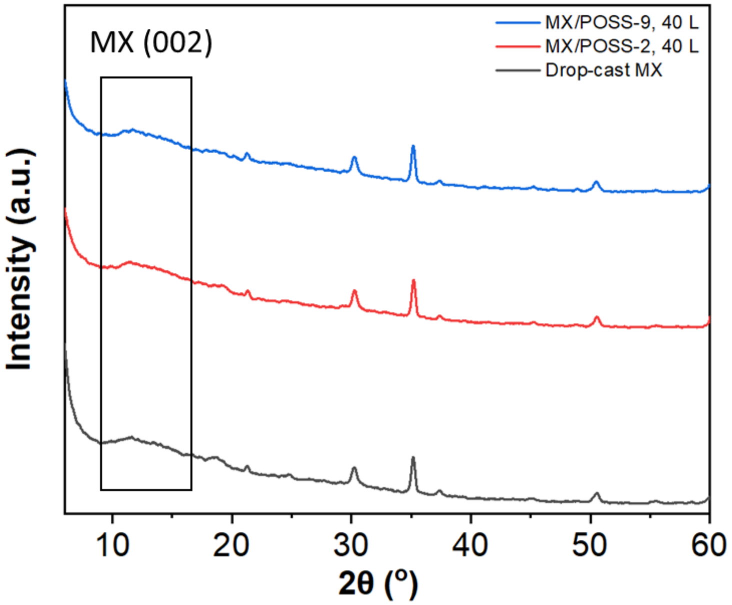

Figure S10. XRD pattern for drop-cast MXene, MX/POSS-2, 40L, and MX/POSS-9, 40L.

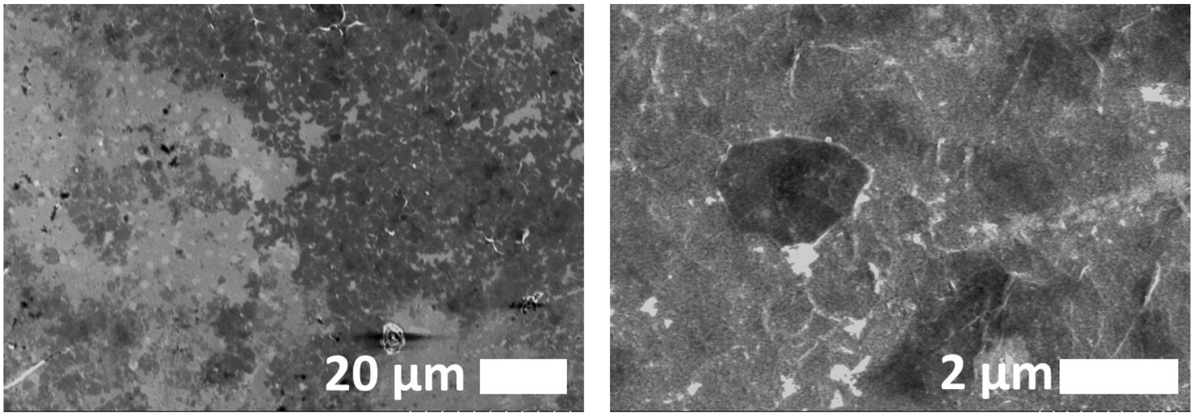

Figure S11. Surface SEM images for drop-cast MXenes on ITO glass.

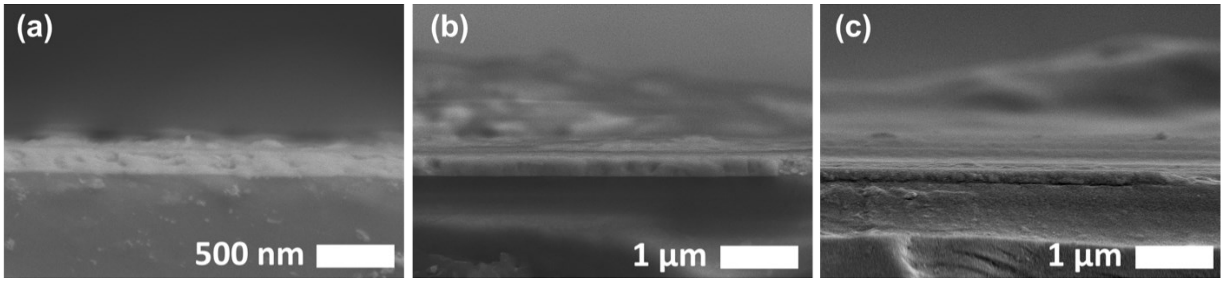

Figure S12. Cross-sectional SEM images for (a) drop-cast MXenes, (b) MX/POSS-2, and (c) MX/POSS-9 on ITO glass.

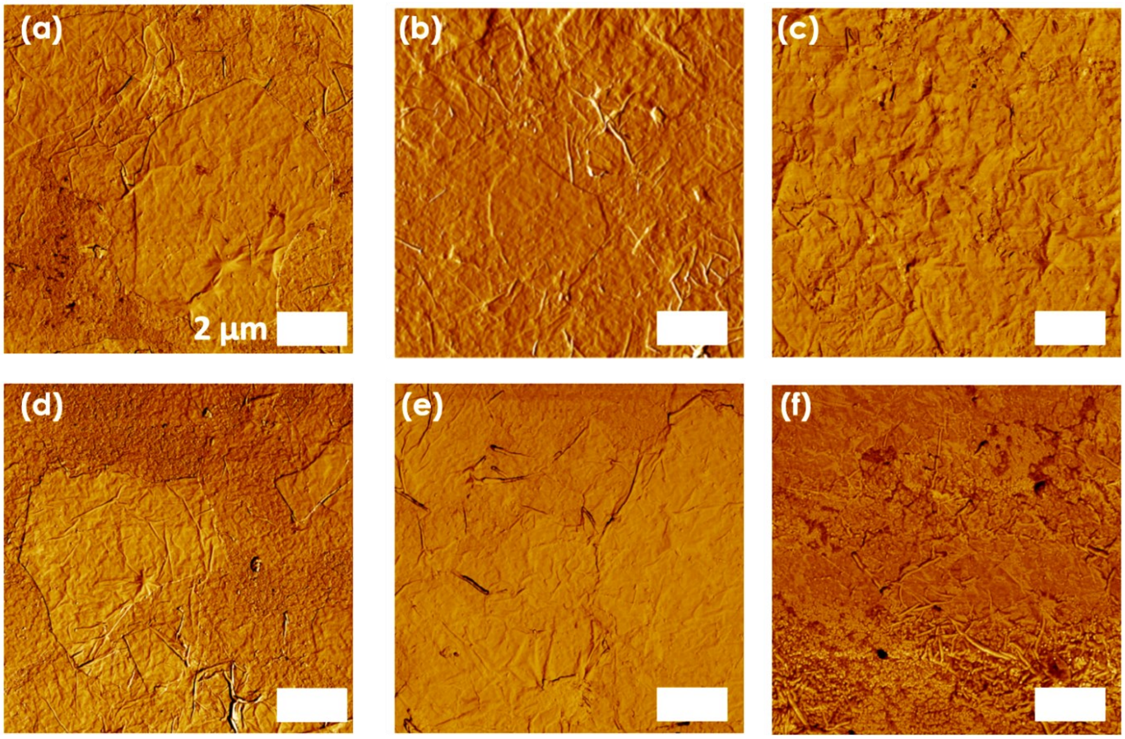

Figure S13. AFM phase images for (a, b, c) MX/POSS-2 and (d, e, f) MX/POSS-9 multilayers of (a, d) 20 layers, (b, e) 30 layers, and (c, f) 40 layers. The Z-axis is 10º for all images. The scale bar of 2 µm applies to all panels.

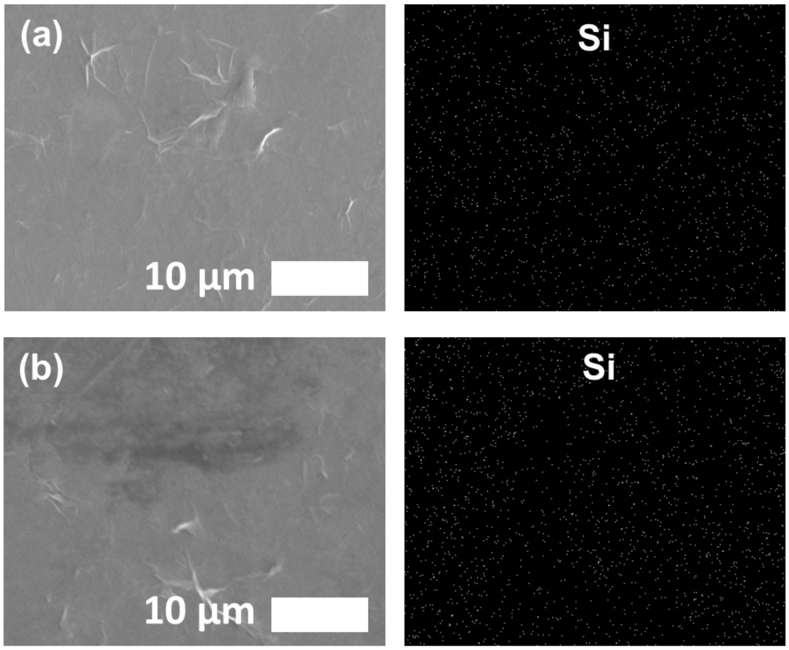

Figure S14. Surface SEM images with corresponding EDS mapping of Si for (a) MX/POSS-2, 40L, and (b) MX/POSS-9. The scale bar is the same for all images.

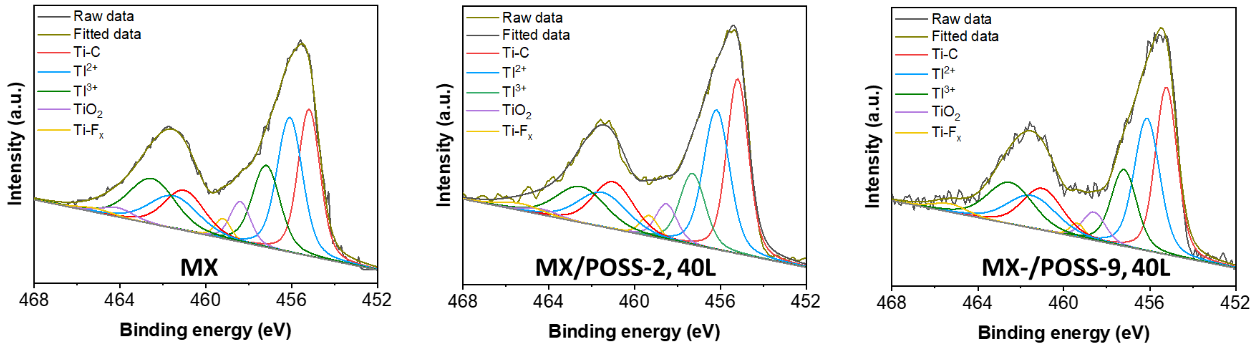

Figure S15. High resolution Ti2p deconvoluted peaks for pure MX, MX/POSS-2, 40L, and MX/POSS-9, 40L films.

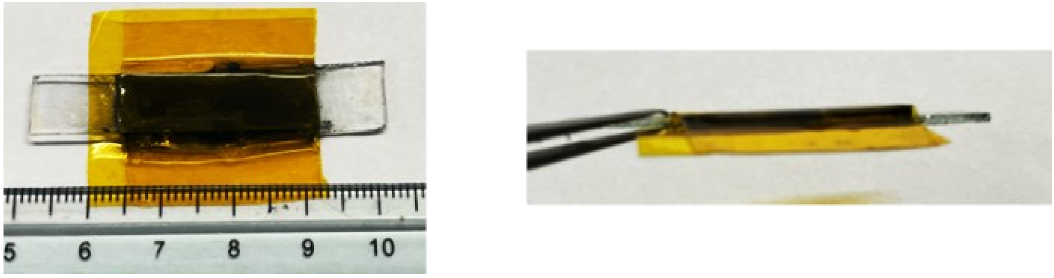

Figure S16. Photos of the 2-eelctrode symmetric supercapacitors fabricated in this work.

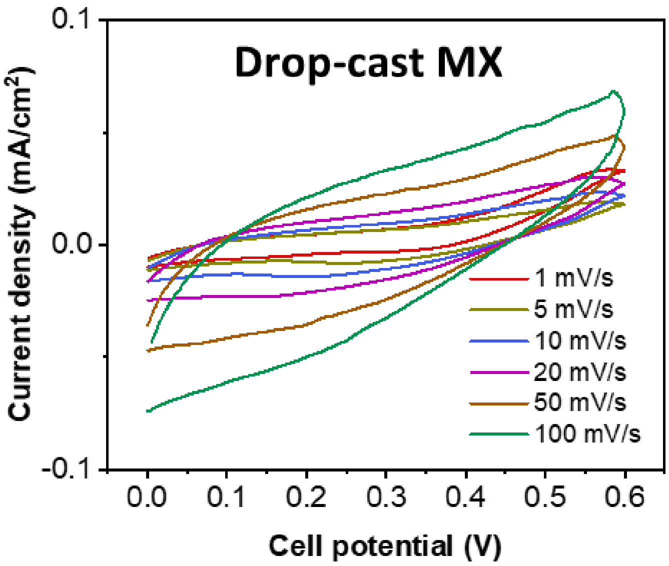

Figure S17. Cyclic voltammograms for drop-cast MX at different scan rates.

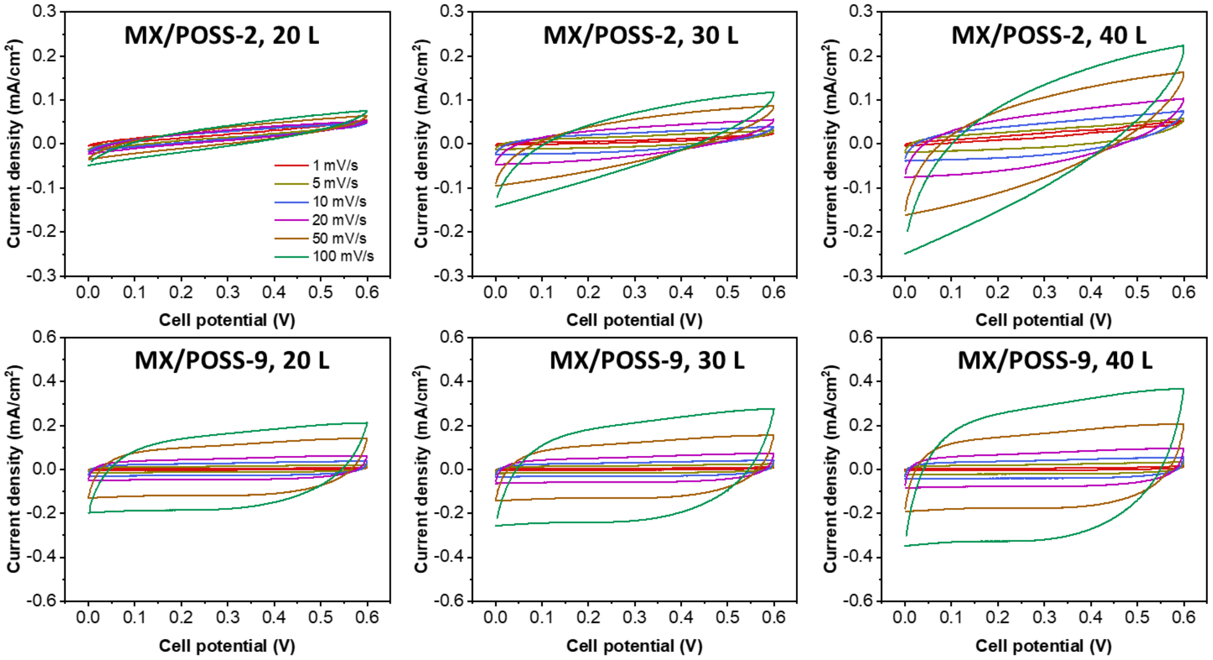

Figure S18. Cyclic voltammograms for multilayered MX/POSS-2 and MX/POSS-9 electrodes at different scan rates.

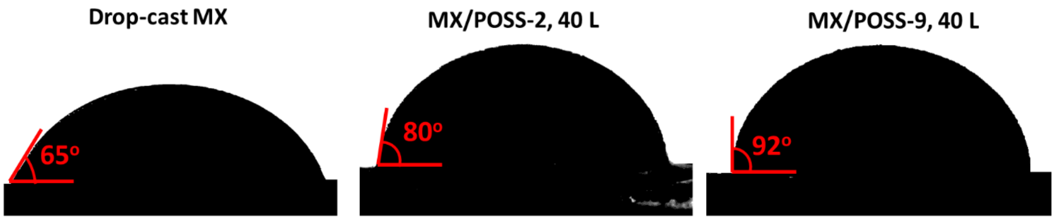

Figure S19. Contact angle measurement images (in black and white) for drop-cast MX, MX/POSS-2, 40L, and MX/POSS-9, 40L films using an aqueous 40 μl droplet.

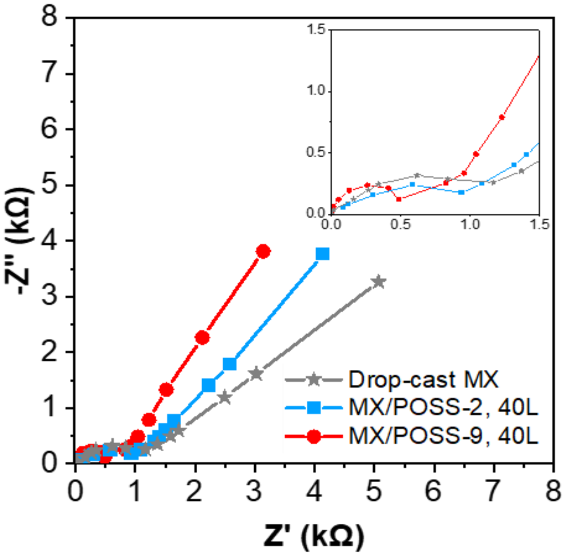

Figure S20. Nyquist plot for drop-cast MX, MX/POSS-2, 40L, and MX/POSS-9, 40L films.

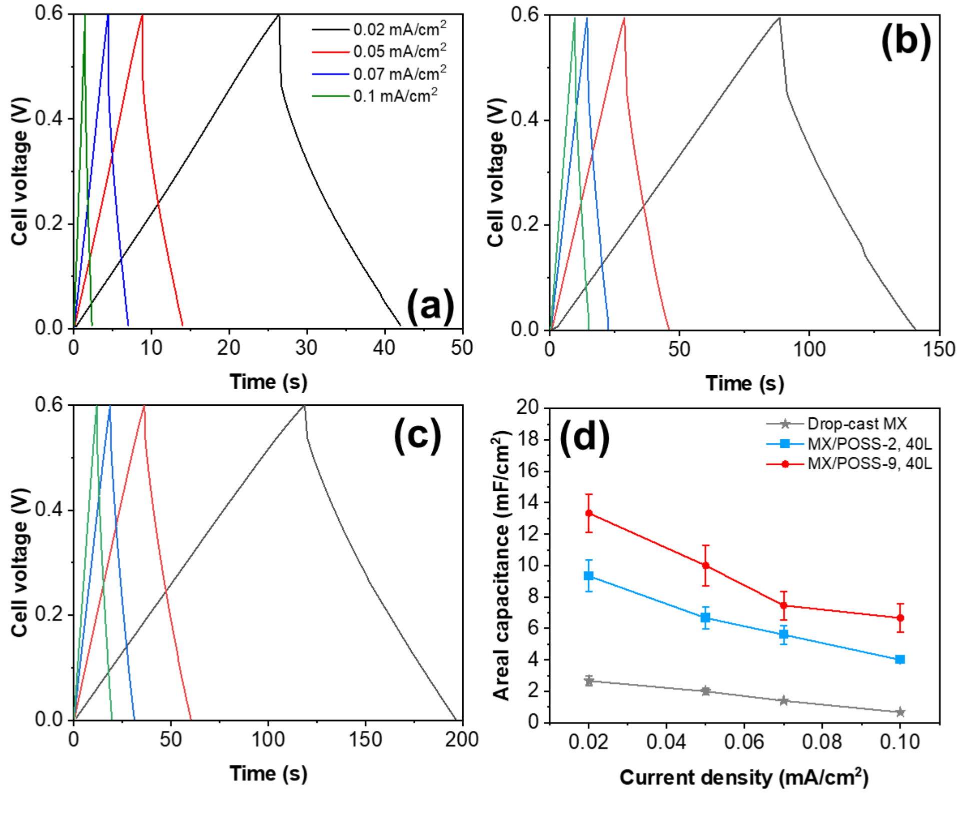

Figure S21. Galvanostatic charge-discharge experiments at variant current densities (0.02 – 0.1 mA/cm^2) for (a) drop-cast MX film, (b) MX/POSS-2, 40 L, and (c) MX/POSS-9, 40 L. (d) Areal capacitance *vs.* current density for drop-cast MX, MX/POSS-2, 40 L, and MX/POSS-9, 40 L.

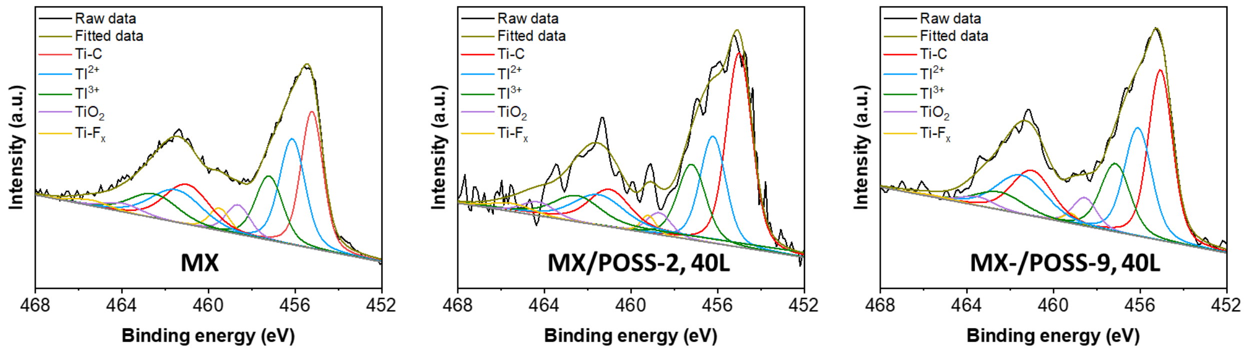

Figure S22. High resolution Ti2p deconvoluted peaks for drop-cast MX, MX/POSS-2, 40L, and MX/POSS-9, 40L films, after 10,000 electrochemical cyclings.

Table S1. Hydrodynamic diameter and ζ – potential for MXenes, POSS nanoparticles, and their mixtures.

Sample	MX	POSS-2	POSS-9	MX/POSS-2	MX/POSS-9
Hydrodynamic diameter (nm)	1600 ± 390	70 ± 6	140 ± 40	1320 ± 40	1290 ± 190
ζ - potential (mV)	- 27 ± 1	30 ± 2	41 ± 2	10 ± 1	19 ± 1

Table S2. Chemical composition of MXene, MX/POSS-2, and MX/POSS-9 films as measured using XPS.

Samples/Elements	MX	MX/POSS-2, 40 L	MX/POSS-9, 40 L
C (at%)	51.9	57.9	59.6
O (at%)	20.9	16.8	17.1
Ti (at%)	18.9	13.5	10.6
F (at%)	6.6	2.8	2.3
Cl (at%)	1.7	1.0	0.9
Si (at%)	-	6.2	5.9
N (at%)	-	1.1	1.9
Br (at%)	-	1.1	1.7

Table S3. Composition of deconvoluted C1s peaks for MXene, MX/POSS-2, and MX/POSS-9 films as measured using XPS.

Component	Binding energy (eV)	FWHM	MX (%)	MX/POSS-2, 40 L (%)	MX/POSS-9, 40 L (%)
C-Ti-T_x^{α}	281.4	0.9	12.0	9.7	9.3
C-Ti-T_x^{α}	282.3	1.9	6.6	3.3	4.0
C-Si	284.1	1.4	-	7.7	10.2
C-C	284.8	1.5	56.0	45.9	42.9
C-O/C-N	286.3	1.4	20.1	28.2	28.6
COO	288.5	1.9	5.5	5.3	5.0

*Tx refers to terminal groups grafted on the MXenes; the bond is asymmetric and as a result it is represented by two peaks.

Table S4. Composition of deconvoluted Ti2p peaks for MXene, MX/POSS-2, and MX/POSS-2 films as measured using XPS.

Component	Binding energy eV	FWHM	MX (%)	MX/POSS-2, 40 L (%)	MX/POSS-9, 40 L (%)
Ti-C	455.1 ($2p_{3/2}$)<br>460.9 ($2p_{1/2}$)	1.2<br>2.2	33.6	35.3	34.8
Ti^{2+}	455.9 ($2p_{3/2}$)<br>461.5 ($2p_{1/2}$)	1.4<br>2.7	31.8	31.8	32.5
Ti^{3+}	457.1 ($2p_{3/2}$)<br>462.6 ($2p_{1/2}$)	1.5<br>2.5	24.5	23.0	23.1
TiO_2	458.5 ($2p_{3/2}$)<br>464.1 ($2p_{1/2}$)	1.1<br>1.9	7.1	6.8	6.3
Ti-F_x	459.3 ($2p_{3/2}$)<br>465.1 ($2p_{1/2}$)	0.9<br>1.8	3.0	3.1	3.3

Table S5. Composition of deconvoluted Ti2p peaks for MXene, MX/POSS-2, and MX/POSS-9 films, after 10,000 of electrochemical cycling.

Component	Binding energy eV	FWHM	MX (%)	MX/POSS-2, 40 L (%)	MX/POSS-9, 40 L (%)
Ti-C	455.1 ($2p_{3/2}$)<br>460.9 ($2p_{1/2}$)	1.2<br>2.2	34.2	35.6	35.1
Ti^{2+}	455.9 ($2p_{3/2}$)<br>461.5 ($2p_{1/2}$)	1.4<br>2.7	32.4	32.8	33.9
Ti^{3+}	457.1 ($2p_{3/2}$)<br>462.6 ($2p_{1/2}$)	1.5<br>2.5	21.0	20.4	20.2
TiO_2	458.5 ($2p_{3/2}$)<br>464.1 ($2p_{1/2}$)	1.1<br>1.9	9.5	7.3	7.5
Ti-F_x	459.3 ($2p_{3/2}$)<br>465.1 ($2p_{1/2}$)	0.9<br>1.8	2.9	3.9	3.3

Table S6. Areal, volumetric, and gravimetric capacitance for thin film supercapacitors (MXene = $Ti_3C_2T_x$).

Sample	Thickness (nm)	Electrolyte	Areal capacitance (Volumetric capacitance)	Capacitance retention	Testing setup
MXene-SC[5]	50	PVA/H_3PO_4	7.5 mF/cm^2 at 10 mV/s (1500 F/cm^3)	N/A	2-electrode
MXene-LB[6]	65	PVA/H_2SO_4	1.2 mF/cm^2 at 3.5 µA/cm^2 (185 F/cm^3 at 0.5 A/cm^3)	90% after 500 cycles at 20.83 µA/cm^2	2-electrode
MXene/PDADMA LbL[7]	63	1M H_2SO_4	4.7 mF/cm^2 at 10 mV/s (746 F/cm^3)	N/A	3-electrode
MXene/NH_2-MXene LbL[7]	63	1M H_2SO_4	13 mF/cm^2 at 10 mV/s (2,064 F/cm^3)	45.8% after 1,000 cycles at 0.025 mA/cm^2	3-electrode
$Ti_3C_2T_x$/RuO_2/AgNW[8]	270	PVA/KOH	23.3 mF/cm^2 at 1 mV/s (864.2 F/cm^3)	90 % after 10,000 cycles at 10 mV/s	2-electrode
$Ti_3C_2T_x$ -LP[9]	650	PVA/H_2SO_4	8.84 mF/cm^2 at 0.25 mA/cm^2 (136 F/cm^3)	70 % after 5,000 cycles at 1 mA/cm^2	2-electrode
rGO, LbL[10]	10	PVA/H_3PO_4	0.394 mF/cm^2 at 0.3 mA/cm^2 (394 F/cm^3)	93 % after 1,500 cycles at 0.3 mA/cm^2	2-electrode
rGO/CNT, LbL[11]	200	PVA/H_3PO_4	2.63 mF/cm^2 at 2 µA/cm^2 (394 F/cm^3)	92% after 3,000 cycles at 5 µA/cm^2	2-electrode
Drop-cast MX (this work)	300	PVA/H_2SO_4	2.2 ± 0.3 mF/cm^2 at 10 mV/s (~ 73.3 F/cm^3 or ~ 88 F/g)	67 % after 10,000 at 20 mV/s	2-electrode
MX/POSS-2, 40L (this work)	390	PVA/H_2SO_4	11.5 ± 1.2 mF/cm^2 at 10 mV/s (~ 295 F/cm^3 or ~ 460 F/g)	81 % after 10,000 at 20 mV/s	2-electrode
MX/POSS-9, 40L (this work)	230	PVA/H_2SO_4	15.2 ± 1.0 mF/cm^2 at 10 mV/s (~ 661 F/cm^3 or ~ 608 F/g)	91 % after 10,000 at 20 mV/s	2-electrode

References

[1] Mori, H.; Lanzendörfer, M.G.; Müller, A.H.E.; Klee, J.E. Silsesquioxane-Based Nanoparticles Formed via Hydrolytic Condensation of Organotriethoxysilane Containing Hydroxy groups. *Macromolecules* **2004**, *37* (14), 5228-5238, DOI: 10.1021/ma035482o

[2] Gunawidjaja, R.; Huang, F.; Gumenna, M.; Klimenko, N.; Nunnery, G. A.; Shevchenko, V.; Tannenbaum, R.; Tsukruk, V. V. Bulk and Surface Assembly of Branched Amphiphilic Polyhedral Oligomer Silsesquioxane Compounds. *Langmuir* **2009,** *25* (2), 1196-1209, DOI: 10.1021/la803182n

[3] Shevchenko, V. V.; Gumenna, M.; Lee, H.; Klimenko, N.; Stryutsky, O.; Trachevsky, V.; Korolovych, V.; Tsukruk, V. V. Reactive Amphiphilic Aprotic Ionic Liquids Based on Functionalized Oligomeric Silsesquioxanes. *Bull. Chem. Soc. Jpn.* **2021**, *94* (9), 2263-2271, DOI: 10.1246/bcsj.20210211

[4] Mathis, T. S.; Maleski, K.; Goad, A.; Sarycheva, A.; Anayee, M.; Foucher, A. C.; Hantanasirisakul, K.; Shuck, C. E.; Stach, E. A.; Gogotsi, Y. Modified MAX Phase Synthesis for Environmentally Stable and Highly Conductive Ti_3C_2 MXene. *ACS Nano* **2021**, *15* (4), 6420-6429, DOI: 10.1021/acsnano.0c08357

[5] Jiang, Q.; Kurra, N.; Maleski, K.; Lei, Y.; Liang, H.; Zhang, Y.; Gogotsi, Y.; Alshareef, H. N. On-Chip MXene Microsupercapacitors for AC-Line Filtering Applications. *Adv. Energ. Mater.* **2019***, 9* (26), 1901061, DOI: 10.1002/aenm.201901061

[6] Fan, L.; Wen, P.; Zhao, X.; Zou, J.; Kim, F. Langmuir–Blodgett Assembly of $Ti_3C_2T_x$ Nanosheets for Planar Microsupercapacitors. *ACS Appl. Nano Mater.* **2022**, *5* (3), 4170-4179, DOI: 10.1021/acsanm.2c00103.

[7] Echols, I. J.; Yun, J.; Cao, H.; Thakur, R. M.; Sarmah, A.; Tan, Z.; Littleton, R.; Radovic, M.; Green, M. J.; Lutkenhaus, J. L. Conformal Layer-by-Layer Assembly of $Ti_3C_2T_z$ MXene-Only Thin Films for Optoelectronics and Energy Storage. *Chem. Mater.* **2022**, *34* (11), 4884-4895, DOI: 10.1021/acs.chemmater.1c04394

[8] Li, H.; Li, X.; Liang, J.; Chen, Y. Hydrous RuO2-Decorated MXene Coordinating with Silver Nanowire Inks Enabling Fully Printed Micro-Supercapacitors with Extraordinary Volumetric Performance. *Adv. Energ. Mater.* **2019***, 9* (15), 1803987, DOI: 10.1002/aenm.201803987

[9] Hu, H.; Hua, T. An Easily Manipulated Protocol for Patterning of MXenes on Paper for Planar Micro-Supercapacitors. *J. Mater. Chem. A* **2017***, 5* (37), 19639-19648, DOI: 10.1039/C7TA04735E

[10] Yoo, J. J.; Balakrishnan, K.; Huang, J.; Meunier, V.; Sumpter, B. G.; Srivastava, A.; Conway, M.; Mohana Reddy, A. L.; Yu, J.; Vajtai, R.; Ajayan, P. M. Ultrathin Planar Graphene Supercapacitors. *Nano Lett.* **2011**, *11* (4), 1423-1427, DOI: 10.1021/nl200225j

[11] Moon, G. D.; Joo, J. B.; Yin, Y. Stacked Multilayers of Alternating Reduced Graphene Oxide and Carbon Nanotubes for Planar Supercapacitors. *Nanoscale* **2013**, *5* (23), 11577-11581, DOI: 10.1039/C3NR04339H
